# Clonality assessment and detection of clonal diversity in classic Hodgkin lymphoma by next-generation sequencing of immunoglobulin gene rearrangements

**DOI:** 10.1038/s41379-021-00983-8

**Published:** 2021-12-03

**Authors:** Diede A. G. van Bladel, Michiel van den Brand, Jos Rijntjes, Samhita Pamidimarri Naga, Demi L. C. M. Haacke, Jeroen A. C. W. Luijks, Konnie M. Hebeda, J. Han J. M. van Krieken, Patricia J. T. A. Groenen, Blanca Scheijen

**Affiliations:** 1 Department of Pathology, Radboud University Medical Center, Nijmegen, The Netherlands; 2 Radboud Institute for Molecular Life Sciences, Nijmegen, The Netherlands; 3 Pathology-DNA, Rijnstate Hospital, Arnhem, The Netherlands; 4 Department of Medical Oncology, Radboud University Medical Center, Nijmegen, The Netherlands

**Keywords:** Immunogenetics, PCR-based techniques

## Abstract

Clonality analysis in classic Hodgkin lymphoma (cHL) is of added value for correctly diagnosing patients with atypical presentation or histology reminiscent of T cell lymphoma, and for establishing the clonal relationship in patients with recurrent disease. However, such analysis has been hampered by the sparsity of malignant Hodgkin and Reed-Sternberg (HRS) cells in a background of reactive immune cells. Recently, the EuroClonality-NGS Working Group developed a novel next-generation sequencing (NGS)-based assay and bioinformatics platform (ARResT/Interrogate) to detect immunoglobulin (IG) gene rearrangements for clonality testing in B-cell lymphoproliferations. Here, we demonstrate the improved performance of IG-NGS compared to conventional BIOMED-2/EuroClonality analysis to detect clonal gene rearrangements in 16 well-characterized primary cHL cases within the IG heavy chain (IGH) and kappa light chain (IGK) loci. This was most obvious in formalin-fixed paraffin-embedded (FFPE) tissue specimens, where three times more clonal cases were detected with IG-NGS (9 cases) compared to BIOMED-2 (3 cases). In total, almost four times more clonal rearrangements were detected in FFPE with IG-NGS (*N* = 23) as compared to BIOMED-2/EuroClonality (*N* = 6) as judged on identical IGH and IGK targets. The same clonal rearrangements were also identified in paired fresh frozen cHL samples. To validate the neoplastic origin of the detected clonotypes, IG-NGS clonality analysis was performed on isolated HRS cells, demonstrating identical clonotypes as detected in cHL whole-tissue specimens. Interestingly, IG-NGS and HRS single-cell analysis after DEPArray™ digital sorting revealed rearrangement patterns and copy number variation profiles indicating clonal diversity and intratumoral heterogeneity in cHL. Our data demonstrate improved performance of NGS-based detection of IG gene rearrangements in cHL whole-tissue specimens, providing a sensitive molecular diagnostic assay for clonality assessment in Hodgkin lymphoma.

## Introduction

Classic Hodgkin lymphoma (cHL) is a B-cell neoplasm, characterized by the presence of a small number of malignant cells, called Hodgkin and Reed-Sternberg (HRS) cells, residing in a background of inflammatory cells, including reactive B cells and plasma cells^1,2^. In cHL, the HRS cells represent functionally impaired B lymphocytes that have lost the capacity to express immunoglobulins and many of the B-cell markers, but show CD15 and CD30 expression^3^. Evidence for their B-cell origin has been obtained by the detection of clonal rearrangements of the immunoglobulin genes, i.e., the immunoglobulin heavy (IGH) and light chain gene loci^4–7^. Furthermore, the presence of somatic hypermutation (SHM) within the rearranged variable (V) genes demonstrates that HRS cells originate from (post-)germinal center B lymphocytes^8–10^. Although most cHL cases are diagnosed based on clinical presentation and histopathology, the molecular detection of clonal immunoglobulin (IG) gene rearrangements is helpful for diagnosing cases with a more atypical presentation and a histology similar to T cell lymphoma, or for clonal comparison in patients with recurrent disease^11,12^.

Currently, the gold standard for clonality testing of lymphomas is the BIOMED-2/EuroClonality assay developed and validated by the EuroClonality Consortium^13–16^. For assessing B-cell clonality, multiplex PCR reactions are performed for the detection of IGH and kappa light chain (IGK) gene rearrangements, followed by fragment analysis using GeneScan or heteroduplex analysis. The applicability of the BIOMED-2/EuroClonality assay in cHL has been evaluated in different studies, demonstrating B-cell clonality in about 50% of formalin-fixed paraffin-embedded (FFPE) cHL tissue specimens^7,17–23^. Although the combination of both IGH and IGK rearrangement analysis, including incomplete rearrangements not undergoing SHM, has certainly enhanced the detection rate of clonal rearrangements in cHL, more sensitive techniques can improve clonality assessment in cHL.

Recently, a next-generation sequencing (NGS)-based clonality assay for detection of IG gene rearrangements (IG-NGS) has been developed by the EuroClonality-NGS Working Group^24^. In this assay, new primers have been designed for complementary IG gene targets that will result in shorter amplicon length, particularly for the incomplete IGH targets as well as IGK targets. This enables better clonality detection in genomic DNA from FFPE tissue specimens, especially samples with inferior DNA quality. Another advantage of IG-NGS is the immediate sequence availability of the identified clonotypes, both of the malignant clone as well as the non-neoplastic background B cells. This will provide more reliable information to detect minor clones in a background of polyclonal B cells and assessing the clonal relationship of multiple B-cell malignancies within the same patient.

In this study, we compared the performance of NGS-based clonality assessment in cHL to the conventional BIOMED-2/EuroClonality assay to identify clonal IGH and IGK gene rearrangements in whole-tissue specimens, including both FFPE and fresh frozen (FF) tissue. We demonstrate improved performance of the IG-NGS assay to detect B-cell clonality in both types of tissue specimens. Furthermore, our data reveal evidence for biclonality in some cHL tumor samples, indicating that co-existence of multiple malignant clones and clonal diversity can occur in this type of lymphoma.

## Materials and methods

### Selection of cHL and reactive lymphoid tissue samples

This study was performed with 16 lymph node biopsies of patients diagnosed with cHL according to the 2017 revised 4th edition of the World Health Organization classification^25^, for which both FFPE and FF tissue material was available^18^ (Table 1 and Supplementary Table S1). IG-NGS was analyzed on all 16 cases, both FFPE and FF, while BIOMED-2/EuroClonality analysis could only be performed on 15 FFPE cases, due to limited DNA yield for case 13, but included all 16 FF cases. For clone definition and identification, available IG-NGS clonality data of 30 reactive lymphoproliferative samples was analyzed (Supplementary Table S2)^26^. All cHL samples were retrieved from the local archive of the Department of Pathology at Radboud University Medical Center, and collected in accordance with the declaration of Helsinki. This study received approval of the institutional CMO review board (Approval No. 2020-6390).

**Table 1:** Characteristics of classic Hodgkin lymphoma FFPE samples in study cohort.

Case	Diagnosis	Gender	Age at diagnosis (yr)	% HRS cells	% Total B cells	% HRS cells of total B cells	% T cells	EBV status
1	NSHL	M	14	5	40	11	20	Negative
2	NSHL	M	14	5	30	14	60	Negative
3	MCHL	F	14	5	25	17	40	Negative
4	NSHL	F	15	5	10	33	50	Negative
5	NSHL	F	17	5	15	25	30	Positive
6	NSHL	M	22	10	15	40	60	Negative
7	MCHL	M	22	1	30	3	60	Positive
8	NSHL	M	33	3	20	13	70	Positive
9	NSHL	M	38	3	10	23	80	Positive
10	MCHL	M	47	5	30	14	50	Negative
11	NSHL	M	76	5	10	33	60	Negative
12	NSHL	F	19	5	15	25	50	Negative
13	MCHL	M	44	2	20	9	50	Positive
14	MCHL	M	32	15	25	38	60	Negative
15	NSHL	M	61	3	25	11	40	Negative
16	NSHL	M	67	15	20	43	50	Positive

The fraction of HRS cells, background B cells and T cells are estimated based on HE and CD30 (% HRS cells), CD79A (B cells), or CD2/CD3 (T cells) staining of FFPE tissue sections. Epstein-Barr virus (EBV) status was determined by Epstein-Barr encoding region (EBER) in situ hybridization. *NSHL* nodular sclerosis HL, *MCHL* mixed cellularity HL; *yr* years.

### Clonality assessment by next-generation sequencing

Detection of IGH and IGK gene rearrangements by next-generation sequencing (IG-NGS) was performed as recently described^24^. In short, three multiplex PCR reactions were performed with 40–50 ng input DNA for the detection of IG gene rearrangements, which involved variable (V), diversity (D), joining (J), kappa deleting element (KDE), and the recombination signal sequence (RSS) gene segments of the following five targets: IGHV-IGHD-IGHJ (FR3), IGHD-IGHJ, IGKV-IGKJ, IGKV-KDE, and Intron RSS-KDE. After bead purification, library preparation and amplification, the samples were pooled with equal DNA concentrations (corresponding to equimolar amounts) for sequencing on Ion Torrent PGM. The Ion 318™ Chip Kit v2 BC (Ion Torrent™, Thermo Fisher, Waltham, MA, USA) was loaded with 24 or 32 samples per chip. The sequencing results were analyzed and visualized using the bioinformatics pipeline ARResT/Interrogate (http://arrest.tools/interrogate)^27^. The output data defines a clonotype (e.g., V3-9 -1/20/-6 J3), notated as a combination of the 5′ gene (V3-9), nucleotides deleted from the 5′ gene (-1), the added nucleotides at the junction including the D-segment if present (20), nucleotides deleted from the 3′ gene (-6), and the 3′ gene (J3)^28^. Data were considered reliable with at least a minimum amount of 1000 reads for IGHV-IGHD-IGHJ (mean: 34,917 reads) and IGKV-IGKJ (mean: 12,032 reads), and 500 reads for IGHD-IGHJ (mean: 31,242 reads), IGKV-KDE (mean: 4201 reads), and Intron RSS-KDE (mean: 2869 reads). For the assessment of B-cell clonality, each DNA sample (extracted as independent isolates from FF and FFPE specimens of the same cHL tumor tissue) was analyzed in duplicate by multiplex PCR and NGS to rule out misinterpretation or artifacts, and validate less-abundant clonotypes. Only clonotypes that are detected with a dominant abundancy above threshold in duplicate are considered as clonal rearrangements.

Additional information regarding ‘Materials and Methods’, including the strategy for clone identification, enrichment of HRS cells and single-cell analysis are provided in the Supplementary information.

## Results

### Clonality detection of selected cHL specimens by conventional BIOMED-2/EuroClonality assay

In this study 16 classic Hodgkin lymphoma (cHL) cases were included, of which both archival FF and FFPE tissue specimens were available (Table 1 and Supplementary Table S1)^18^. Eleven cases were of the nodular sclerosing subtype (NSHL), of which four cases (36%) were Epstein-Barr virus (EBV)-positive, and five cases were diagnosed as mixed cellularity HL (MSHL), with two EBV-positive cases (40%). The average percentage of malignant HRS cells in total tissue as judged by CD30 immunohistochemical staining varied between 1% and 15%, and the fraction of HRS cells related to total B-cell counts ranged from 3% to 43% (Table 1 and Supplementary Fig. S3). First, conventional BIOMED-2/EuroClonality was performed on  15 FFPE cases, for which sufficient DNA was available, and 16 FF cases.. Clonal IG gene rearrangements for at least one of the IG targets were detected 10 out of 16 FF samples (63%) and 3 out of 15 FFPE samples (20%) (Table 2 and Supplementary Table S3). The remaining six FF samples showed a polyclonal pattern (34%), while this was observed in 10 out 15 FFPE samples (67%), of which seven displayed nonevaluable results on at least one of the IG targets. Two FFPE samples (case 3 and 10; 13%) yielded no interpretable results on all IG targets, due to the inferior DNA quality (Supplementary Table S3). Notably, in none of the 16 cases clonal IGKV-IGKJ gene rearrangements were detected with the BIOMED-2/EuroClonality assay, due to suboptimal detection of minor clonal rearrangements in the IGKV-IGKJ GeneScan pattern, which displayed polyclonal background over three narrow Gaussian curves.

**Table 2:** Summary clonality analysis in classic Hodgkin lymphoma by BIOMED-2/GeneScan analysis and next-generation sequencing.

			Fresh frozen (FF) tissue		FFPE tissue	
			BIOMED-2 (*N* = 16)	IG-NGS assay (*N* = 16)	BIOMED-2 (*N* = 15*)	IG-NGS assay (*N* = 16)
Molecular conclusion	Clonal		10 (63%)	14 (88%)	3 (20%)	9 (56%)
	Polyclonal					
		4 IG targets	6 (38%)	2 (13%)	3 (20%)	5 (31%)
		<4 IG targets	0 (0%)	0 (0%)	7 (47%)	2 (13%)
	Not evaluable		0 (0%)	0 (0%)	2 (13%)	0 (0%)
Technical description	IGHV-IGHD-IGHJ (FR3)					
		Clonal	5 (31%)	11 (69%)	2 (13%)	3 (19%)
		Polyclonal	11 (69%)	5 (31%)	8 (53%)	11 (69%)
		Not evaluable	0 (0%)	0 (0%)	5 (33%)	2 (13%)
	IGHD-IGHJ					
		Clonal	2 (13%)	4 (25%)	1 (7%)	4 (25%)
		Polyclonal	13 (81%)	12 (75%)	8 (53%)	12 (75%)
		Not evaluable	1 (6%)	0 (0%)	6 (40%)	0 (0%)
	IGKV-IGKJ					
		Clonal	0 (0%)	6 (38%)	0 (0%)	5 (31%)
		Polyclonal	16 (100%)	10 (63%)	13 (87%)	10 (63%)
		Not evaluable	0 (0%)	0 (0%)	2 (13%)	1 (6%)
	IGKV/Intron RSS-KDE					
		Clonal	7 (44%)	10 (63%)	3 (20%)	7 (44%)
		Polyclonal	9 (56%)	6 (38%)	3 (20%)	7 (44%)
		Not evaluable	0 (0%)	0 (0%)	9 (60%)	2 (13%)

Molecular conclusion per case (upper panel) and technical description per IG target (lower panel) are shown for BIOMED-2/GeneScan and IG-NGS analysis with fresh frozen (FF) and FFPE tissue. For BIOMED-2, the results of IGHV-IGHD-IGHJ only indicate FR3, but exclude FR1 and FR2 analysis. For polyclonal cases, both detection of polyclonal patterns on all 4 IG targets and on less than 4 targets (remaining targets were not evaluable) are shown.

*Not performed for one FFPE sample (case 13) due to limited availability of DNA.

### Detection of clonal immunoglobulin gene rearrangements in cHL by IG-NGS

**Fig. 1:**
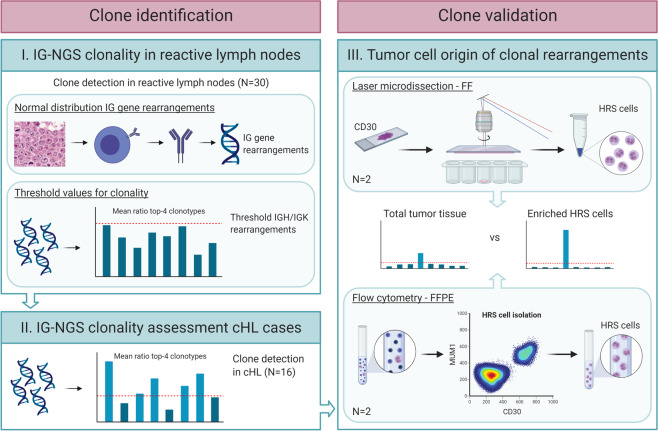
Graphical summary of the workflow for clone identification and validation in classic Hodgkin lymphoma. For clone identification in classic Hodgkin lymphoma (cHL), first the relative frequencies of the most-abundant clonotypes in non-malignant reactive lymph nodes (*N* = 30) were determined by next-generation sequencing of immunoglobulin gene rearrangements (IG-NGS) (Supplementary Fig. S1). Here, clonality analysis was performed in duplicate, followed by calculation of the mean ratio of the top-4 overlapping clonotypes identified in duplicate in each sample, for each of the immunoglobulin heavy chain (IGH) and immunoglobulin kappa light chain (IGK) targets, including both complete and incomplete IG gene rearrangements. Based on the maximum ratio of the overlapping clonotypes in the reactive lymph nodes, a threshold value for clonality was defined for each of the targets. The established threshold values were used to define clonality in the 16 cHL cases. For clone validation, laser microdissection and flow cytometry were used to enrich the Hodgkin Reed-Sternberg (HRS) cells from the tumor-tissue specimen of two cHL cases, and these HRS cell fractions were subjected to IG-NGS clonality analysis to validate these as neoplastic clones.

To define clonality in cHL by IG-NGS, we reasoned that it would be important to identify the clone frequencies of abundant clonotypes in non-malignant reactive lymphoproliferative samples, which would define thresholds above which potentially malignant clonotypes could be identified. Furthermore, it would be essential to demonstrate that identified clonotypes in cHL whole-tissue specimens corresponded to IG gene rearrangements of the corresponding HRS cells, and not reactive B-cell clones. To this end, we established a specific workflow procedure as summarized in Fig. 1.

First, the normal distribution of IG gene rearrangements for all five targets (framework-3 (FR3) IGHV-IGHD-IGHJ, IGHD-IGHJ, IGKV-IGKJ, IGKV-KDE, and Intron RSS-KDE) was established in a cohort of reactive lymphoid tissue specimens (*N* = 30; RLN1-RLN30) (Supplementary Table S2). Here, we focused on the detection of identical clonotypes in two independent IG-NGS assays and sequence runs derived from one reactive tissue sample. By calculating the mean ratio of the top 4 overlapping clonotypes compared to the background clonotypes, the relative abundances of the most dominant clonotypes were defined for each target (Supplementary Fig. S1). Secondly, threshold ratio values were determined for each target in the reactive lymphoproliferative samples by comparing frequencies of the abundant clonotypes relatively to the minor clonotypes (Supplementary Fig. S4). Threshold values for clonality were set as such that all reactive lymphoproliferative samples did not exceed this ratio, resulting in the values 2.5 for IGHV-IGHD-IGHJ (FR3), 4.5 for IGHD-IGHJ, 4 for IGKV-IGKJ, and 2.5 for IGKV-KDE clonotypes. Based on the limited diversity in Intron RSS-KDE rearrangements, the threshold was based on the relative mean abundance of all rearrangements involving KDE, and determined on 4.5% (no ratio calculation).

Next, NGS-based clonality assessment was performed on each of the 16 cHL cases in duplicate, for both FF and FFPE samples. Taking the established thresholds for clone definition, we could detect clonal rearrangements in at least one of the IG targets in 14 out of 16 FF cases (88%) and 9 out of 16 FFPE cases (56%) with IG-NGS (Table 2, Supplementary Tables S3 and S4, and Supplementary Fig. S5). FF yielded interpretable results for all samples, while FFPE remained problematic in two DNA samples (case 10 and 13), which yielded too low read counts for clone identification on multiple targets, due to inferior DNA quality (Supplementary Table S3). Identical clonal rearrangements were detected in the paired FFPE and FF tissues with clonal IG gene rearrangements, demonstrating a good correlation. Alignment of the obtained IGHV and IGKV nucleotide sequences of the identified cHL clonotypes to their germline sequences showed evidence for SHM within the malignant cHL clones (Supplementary Fig. S6).

**Fig. 2:**
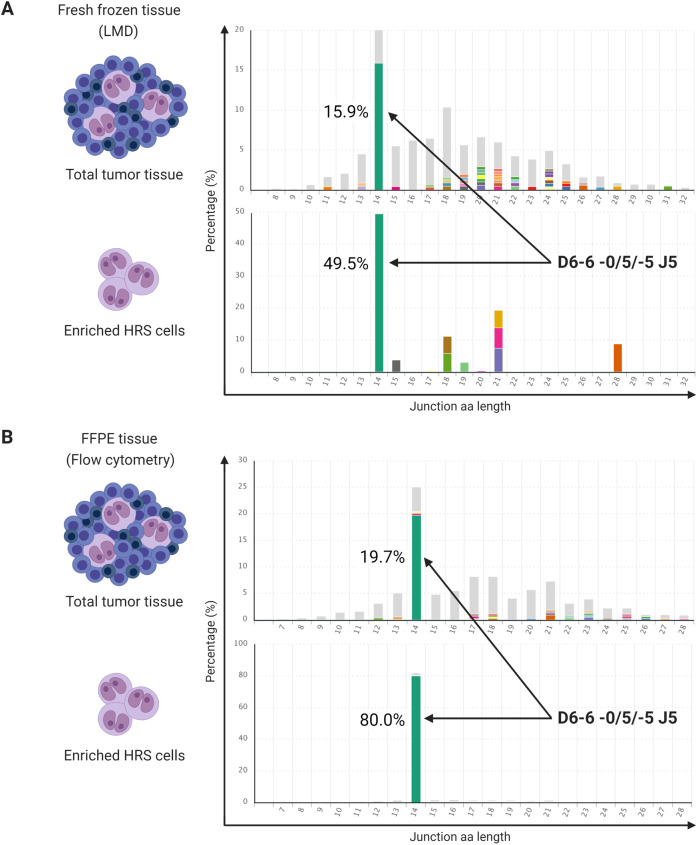
Validation of identified clonotypes in whole-tumor tissue by next-generation sequencing of immunoglobulin gene rearrangements in HRS-enriched cells. NGS-based clonality analysis was performed on DNA isolated from whole-tumor tissue and Hodgkin Reed-Sternberg (HRS)-enriched cell suspensions of a classic Hodgkin lymphoma (cHL) sample (case 16), archived as a fresh frozen (FF) and formalin-fixed paraffin-embedded (FFPE) tissue specimen. The isolation of HRS cells was performed with laser microdissection (LMD) of CD30^+^ cells in FF tissue, and flow cytometric sorting of CD30^+^/MUM1^+^ HRS cells after mild enzymatic digestion of FFPE tissue. Identified clonotypes for target IGHD-IGHJ of FF (**A**) and FFPE (**B**) specimen are shown, in the order whole tissue, followed by HRS-enriched cell fraction.

To actually confirm that the identified clonotypes were of neoplastic cell origin, we enriched the HRS cells in FF and FFPE material of two cHL cases (case 8 and 16) using laser microdissection (LMD) of CD30^+^ HRS cells in FF tissue sections, and flow cytometric isolation of CD30^+^MUM1^+^ HRS cells after mild enzymatic digestion of the paired FFPE tissue specimen, as described by Juskevicius et al.^29^. Genomic DNA was isolated from the HRS-enriched cell fractions of both procedures, and subjected to IG-NGS analysis. In each of the two cHL cases, identical clonotypes were detected in higher abundance in the HRS-enriched cell fractions as compared to whole-tissue specimens, both for FF as well as FFPE tissues (Fig. 2 and Supplementary Table S5). These results confirmed that the clonal IG gene rearrangements and corresponding clonotypes as detected by IG-NGS in cHL are of neoplastic cell origin.

### Improved performance of IG-NGS compared to BIOMED-2/EuroClonality assay for clonality assessment in cHL

**Fig. 3:**
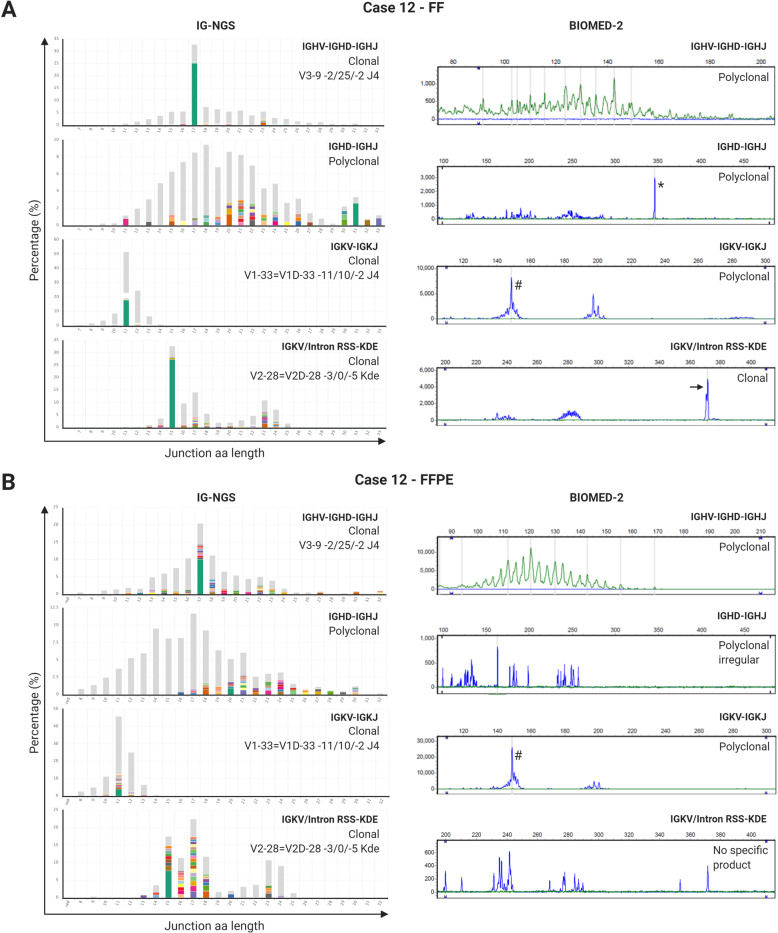
Clonality assessment of classic Hodgkin lymphoma by next-generation sequencing as compared to BIOMED-2/GeneScan assay. The results of clonality analysis for (**A**) fresh frozen (FF) and (**B**) formalin-fixed paraffin-embedded (FFPE) tissue of case 12 are shown for all IG targets (IGHV-IGHD-IGHJ, IGHD-IGHJ, IGKV-IGKJ, and IGKV/Intron RSS-KDE) based on next-generation sequencing of immunoglobulin gene rearrangements (IG-NGS) (left panels) and BIOMED-2/GeneScan assay (right panels). IG-NGS clonality results: junction length in amino acids (junction aa length) is shown on the *x*-axis, the abundancy of clonotypes is shown in percentages on the *y*-axis. Each bar represents a total of all detected clonotypes with the same junction length, each color represents a unique clonotype based on their nucleotide sequence. The 50 most-abundant clonotypes are shown in colors, all other clonotypes are depicted together in gray. Clonotypes are described by the 5′ and 3′ gene annotation and the junctional nucleotide sequence of the rearrangement. BIOMED-2/GeneScan clonality results: clonal product is marked with black arrow for IGKV/Intron RSS-KDE in FFPE sample; ^*^non-specific IGHD-IGHJ non-specific germline product; ^#^IGKV-IGKJ polyclonal signal, the narrow Gaussian curve in the IGKV 1f/6/7-IGKJ size window is due the high homology of the IGKV genes and explains why minor clonal rearrangements can be easily missed.

To evaluate the actual performance of IG-NGS for clonality assessment in cHL, we compared the results of NGS-based clonality analysis with the BIOMED-2/EuroClonality assay (Fig. 3). Notably, with conventional BIOMED-2/EuroClonality analysis, detection of IGHV-IGHD-IGHJ gene rearrangements is based on primers covering FR1, FR2, and FR3 regions. Since in our current IG-NGS assay, no FR1 and FR2 primers were included for detecting IGHV-IGHD-IGHJ gene rearrangements, the comparison involved only the FR3 region. IG-NGS was able to detect clonal rearrangements in 14 cHL cases (88%) as compared to 10 cases (63%) with BIOMED-2/EuroClonality assay, when assessing FF DNA (Table 2). In 11 out of 14 clonal FF samples (79%), clonality was detected on multiple targets with IG-NGS, while this was observed in only 2 out of 10 clonal FF samples (20%) with the BIOMED-2/EuroClonality assay (Table 3 and Supplementary Table S3). In FF, IG-NGS detected clonal rearrangements in six additional samples for target IGHV-IGHD-IGHJ (FR3), two samples for IGHD-IGHJ, six samples for IGKV-IGKJ and three samples for IGKV/Intron RSS-KDE targets as compared to BIOMED-2/EuroClonality assay. Two samples (cases 9 and 13) showed also additional clonal IGKV/Intron RSS-KDE rearrangements with IG-NGS (Table 3), demonstrating the increased sensitivity and specificity of IG-NGS clonality testing in FF tissue. This is due to the improved detection of clonotypes within a polyclonal background, especially for IGKV-IGKJ and IGKV/intron RSS-KDE rearrangements^30^. However, IG-NGS failed to detect an IGHV-IGHD-IGHJ gene rearrangement for case 16, which was identified with conventional BIOMED-2/EuroClonality testing for FR3 using a different IGHV-FR3 forward primer (Table 3). Further evaluation of the FR1 BIOMED-2/GeneScan data of this case showed a shorter amplicon length than expected (173 bp vs ~230–295 bp) (Supplementary Fig. S7). Sanger sequencing of this amplicon revealed a 97 bp deletion within the IGHV3-74 gene, which harbored the annealing site of the IG-NGS FR3 primer, while the sequence of the BIOMED-2 FR3 primer was still present (Supplementary Fig. S7).

**Table 3:** Overview improved performance of NGS-based clonality assessment compared to the BIOMED-2/EuroClonality assay in classic Hodgkin lymphoma.

The clonality results are based on duplicate analysis for FF and FFPE tissue samples for both techniques. All rearrangements are detected in the presence of a polyclonal background. Colors used: green: improved performance IG-NGS (NGS) compared to BIOMED-2/GeneScan (GS), lighter shade indicates improvement (evaluable results), without detection of clonal rearrangement; blue: failed detection of clonotype by IG-NGS compared to BIOMED-2/EuroClonality (see Supplementary Fig. S7); orange: added value of FR1/2 testing, based on the GS results (see Supplementary Table S3). *P* polyclonal, *Pirr* polyclonal irregular pattern, *P_LE* polyclonal_less evaluable, the results are based on less than 4 interpretable IG targets and therefore potential clonal results can be missed, resulting in a molecular conclusion that may be less reliable compared to samples with interpretable results on all 4 IG targets; *R* clonal rearrangement, *C* clonal, *BC* biclonal, *NE* not evaluable, *ND* not done.

^*^IGHD1/2/3/5-IGHJ and IGKV2/4/5 are not evaluable, due to suboptimal DNA quality. ^#^IGHV-IGHD-IGHJ results on the basis of BIOMED-2 FR3 primer only. ^‡^IGHV-IGHD-IGHJ results on the basis of BIOMED-2 FR1, FR2, and FR3 primers. ^†^The second clonal IGHD-IGHJ rearrangement was observed in HRS-enriched FF DNA fraction (see Supplementary Table S5).

In FFPE tissue specimens, IG-NGS detected an even higher number of additional clonal IG gene rearrangements with the BIOMED-2/EuroClonality assay. In total, 9 cases (56%) showed clonal IG gene rearrangements with IG-NGS, while only 3 clonal cases (20%) were detected with BIOMED-2/EuroClonality, and clonality assessment with IG-NGS was often supported by multiple targets (Table 3 and Supplementary Table S3). Similar to the FF sample, one clonal IGHV-IGHD-IGHJ rearrangement could not be detected with IG-NGS, due to a 97 bp deletion (case 16). There was one cHL tumor (case 6) with a clonal IGHV-IGHD-IGHJ gene rearrangement detected in FR2 by the BIOMED-2/EuroClonality assay, which was not identified by the IG-NGS approach since FR2 was not included in the current assay (Table 3). Overall, only one (case 15) out of 16 cHL samples (6%) showed no detectable IG gene rearrangements with both techniques.

### Detection of clonal diversity and intratumoral heterogeneity in cHL by single-cell analysis

Since NGS-based clonality assessment revealed more detailed information regarding the clonal composition of lymphomas, two out of 14 cases (cases 8 and 9; 14%) showed multiple clonal IG gene rearrangements exceeding the allelic capacity of a single B-cell clone. Pathology review provided no indications for a composite lymphoma in both cHL FFPE tissue specimens. Case 8 displayed two unproductive IGHD-IGHJ gene rearrangements (IGHD3-9/IGHJ6 and IGHD1-26/IGHJ5) combined with one productive IGHV-IGHD-IGHJ gene rearrangement (IGHV3-9/IGHD6-13/IGHJ3), implying the presence of two B-cell clones (Supplementary Fig. S8). All three IGH gene rearrangements were identified in both whole-tissue and bulk HRS-isolated cells (Supplementary Table S5). Case 9 showed an even more complex pattern of unrelated IG gene rearrangements. Multiple clonal rearrangements were detected on both IGH (two in-frame IGHV-IGHD-IGHJ rearrangements) and IGK targets (three unrelated IGKV-IGKJ and four IGKV/Intron RSS-KDE rearrangements), suggesting also biclonality (Table 3, Supplementary Table S4, and Fig. S8). These data demonstrate that clonal diversity in cHL can be determined by deep-sequencing of IG gene rearrangements.

**Fig. 4:**
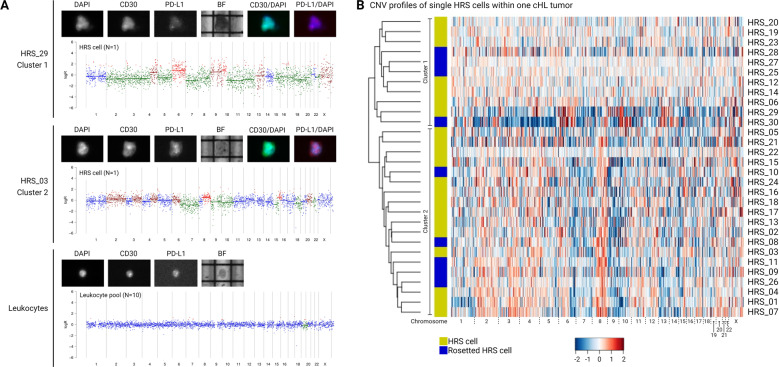
Single-cell copy number variation analysis reveals clonal diversity in classic Hodgkin lymphoma. Single HRS cells, which were obtained after mild enzymatic digestion of formalin-fixed paraffin-embedded tissue specimen (case 9), were isolated with DEPArray digital sorting technology based on CD30 and PD-L1 expression. Subsequently, genomic DNA was isolated and subjected to whole-genome amplification, shallow whole-genome sequencing and copy number analysis. HRS cells are defined as hyperdiploid, CD30^+^ PD-L1^+^ enlarged cells/nuclei. **A** Microscopy images and corresponding copy number variation (CNV) profiles of two representative single HRS cells (one from each cluster) and control leukocyte of the same tissue. The CNV profiles show the genome location according to chromosome position on the *x*-axis and the log-scaled copy number values on the *y*-axis. **B** Unsupervised hierarchical clustering of CNV profiles of 30 HRS cells (21 single HRS cells (yellow) and 9 rosetted HRS cells (blue)) reveals two main clusters (designated with vertical lines), indicating biclonality of the cHL-tumor tissue. The *x*-axis shows the chromosome position and the *y*-axis the CNV profiles of the individual HRS cells.

To confirm in an independent manner the clonal composition of these two cHL cases, we aimed to perform copy number variation (CNV) profiling on CD30^+^PD-L1^+^ HRS single cells isolated from FFPE specimens by DEPArray™ sorting technology^31,32^. This procedure involves whole-genome amplification (WGA) of single cells followed by shallow whole-genome sequencing (sWGS), and subsequent CNV analysis of the individual HRS cells. DNA integrity analysis with quantitative PCR on DNA isolated from the FFPE tissue specimens indicated that only case 9 (QC score: 0.35) fulfilled the quality criteria for the subsequent sWGS approach. Unsupervised hierarchical cluster analysis revealed two separate clusters with distinct CNV profiles of the individual HRS cells (Fig. 4 and Supplementary Figs. S9 and S10). Each cluster showed defined regions with copy number gains and losses, and both populations showed evidence of intraclonal heterogeneity, reminiscent of the genomic instability described for cHL^33,34^. Overall, NGS-based clonality assessment showed improved performance in cHL, including increased sensitivity and specificity of detecting clonal IG gene rearrangements, and providing accurate information on the clonal composition of the lymphoid malignancy.

## Discussion

Detection of IG gene rearrangements for clonality analysis in cHL has remained challenging due to the low frequency of malignant HRS cells in a background of reactive B cells and plasma cells. By direct comparison of NGS-based clonality assessment with the conventional BIOMED-2/GeneScan analysis to detect IG gene rearrangements in undissected cHL-tumor tissue, our study demonstrated a significantly improved performance of the IG-NGS assay. The clonotypes detected by IG-NGS were shown to be of neoplastic cell origin, based on the identification of identical IG gene rearrangements in HRS-enriched cell fractions as compared to whole tissue specimens. Furthermore, our data revealed evidence for biclonality in two cHL cases, which was confirmed by single-cell analysis in one of the cases.

The recently developed IG-NGS clonality assay of the EuroClonality-NGS Working Group set out to improve clonality detection in lymphoid malignancies and has been validated for different mature B-cell lymphoma subtypes, but not yet for cHL^24,26^. Here, we investigated the applicability of the IG-NGS assay to detect clonal IG gene rearrangements in cHL. To define threshold values for tumor clone identification in cHL in the context of this study, clonality data of reactive lymphoproliferative samples without clear indications for EBV positivity were used to establish relative B-cell clone frequencies in non-malignant tissues. Identical clonotypes could be identified in duplicate analysis of the reactive lymphoproliferative samples with slightly increased frequencies compared to less-abundant background clonotypes.

On the basis of our defined thresholds, clonal IG gene rearrangements were detected with IG-NGS in 88% of the FF samples and 56% of the FFPE specimens, often at multiple targets for both IGH and IGK. Since all clonotypes were detected in duplicate in both FF and FFPE, these likely represent true clonal rearrangements and not artifacts. Furthermore, the identified dominant clonotypes showed no overlap between different samples, excluding cross-contamination or index-hopping. As expected, cHL tissues with relative higher frequencies of CD30-positive HRS cells as a proportion of total B cells showed more pronounced dominant clonotypes (Supplementary Fig. S11). Furthermore, a greater number of clonal IG gene rearrangements were detected in FF as compared to FFPE tissue, probably related to a higher genomic DNA integrity in FF material. However, each clonal rearrangement identified in FFPE was also detected in the paired FF cHL sample. In addition, IG-NGS detected clonality at more IG targets and additional cHL cases as compared to the BIOMED-2/EuroClonality assay, demonstrating the additive value of this approach. A recent study analyzed 120 non-Hodgkin lymphoma cases with IG-NGS^26^, where clonal IG gene rearrangements were detected in 96% of the lymphoma cases. This demonstrates that the identification of clonal IG gene rearrangements in cHL with NGS is less efficient (56% in FFPE, 88% in FF), most likely due to the lower fraction of malignant HRS cells within the tumor specimens. Studies in larger cohorts are required to firmly establish the clonality detection rate in cHL.

In three FF and seven FFPE samples, identical IG gene rearrangements were detected in top 3 duplicate clonotypes that did not reach our defined thresholds for clonality, and were indicated as ‘possible minor clones with unknown significance’ (Supplementary Table S4). In most of the FFPE samples, these clonotypes reached the thresholds for clonality in the corresponding FF sample and could therefore, in combination with the estimated tumor cell count, be considered as tumor-related clones for which detection was less efficient. Other minor clones, especially in the presence of a dominant tumor clone, most likely represented reactive B- or plasma cell clones, which in some cases could be related to the EBV status (Table 1). The increased sensitivity and specificity of IG-NGS relates to the smaller amplicons of the IG-targets in the assay in combination with a different primer design compared to the BIOMED-2/EuroClonality assay, the deep sequencing technique, and the information of specific clonotypes with the corresponding nucleotide sequences. Therefore, minor clones can be identified that otherwise would be blurred by the polyclonal background of conventional BIOMED-2/GeneScan analysis. On the other hand, all three frameworks for IGHV-IGHD-IGHJ rearrangements are analyzed in the BIOMED-2/EuroClonality assay, whereas the current IG-NGS assay is focussed on FR3.

Our studies demonstrate the presence of SHM in IGHV and to a lesser extent in IGKV gene regions of the malignant HRS cells (Supplementary Fig. S6), which is in line with previous findings^8,9,35^. Notably, 12 out of 14 cases displayed productive clonal IGHV-IGHD-IGHJ FR3 gene rearrangements, with CDR3 open reading frames ranging from 14 to 32 amino acids (Supplementary Tables S4 and S6). This is in concordance with a previous study, demonstrating that the majority of HRS IG gene rearrangements are functional^9^. However, HRS cells lack expression of the B-cell receptor (BCR), which is most likely due to the absence of active IG gene-specific transcription^9^ and epigenetic silencing of the BCR locus^36^. Nonetheless, IG gene rearrangements in cHL can serve as informative molecular fingerprints for clonality testing of cHL. To confirm that the observed dominant clonotypes in undissected tumor-tissue samples were derived from neoplastic cell origin, HRS cells were enriched by flow cytometry (FFPE tissue) and LMD (FF tissue). IG-NGS clonality testing showed identical clonal gene rearrangements and corresponding clonotypes at a higher abundancy in the isolated HRS-cell fraction as compared to whole-tumor tissue DNA, substantiating their neoplastic cell origin.

Furthermore, our data revealed the presence of multiple clonal rearrangements, which suggests the occurrence of biclonality in cHL (Supplementary Fig. S8). Although true bi- or oligoclonality is an infrequent phenomenon in B-cell lymphoproliferative disorders^37,38^, biclonality has been reported mostly in the context of composite lymphomas^39–41^, and also within single lymphoma subtypes^42,43^ and in immunosuppressed patients^44,45^. It is known that, especially in patients with a history of infectious mononucleosis or immune deficiencies, EBV latent antigens can regulate or mimic different survival and proliferative signaling pathways, and thereby promote lymphomagenesis^46–48^. Since both of our cases with suspected biclonality showed EBV positivity, this might contribute to clonal diversity with the detection of two clonal IGHD-IGHJ and one IGHV-IGHD-IGHJ rearrangement in case 8, and multiple IGK gene rearrangements in case 9, which do not fit within a single B-cell clone (Supplementary Fig. S8). Besides this immunogenetic evidence for the involvement of at least two clones, we also employed single-cell CNV analysis on DEPArray digital sorted HRS cells of case 9, which revealed two clusters of distinct CNV profiles. Each of the two HRS clusters displayed prominent intraclonal heterogeneity, which is likely related to the genetic instability of HRS cells^33,34^, and is in concordance with a recent study of Mangano et al.^31^. Unfortunately, such single-cell analysis could not be performed on case 8, due to the inferior FFPE-DNA quality.

In conclusion, IG-NGS clonality testing is a more accurate and sensitive assay for clonality detection in whole-tumor tissue DNA of cHL samples compared to the conventional BIOMED-2/EuroClonality assay. Additionally, this new IG-NGS based clonality testing will be an ideal tool in clinical diagnostics for recurrent disease, since clonal comparison based on nucleotide sequences and assigned clonotypes is more reliable than comparing fragment lengths, as has been shown in non-Hodgkin lymphoma patients^24,49^. Moreover, this approach allows more detailed analysis on the clonal composition and its diversity that underlies cHL pathogenesis.

## Supplementary information

Supplmentary information.

Supplementary Table S4.

## Data Availability

All next-generation sequencing datafiles related to clonality assessment presented in this study are available from the corresponding author on reasonable request.
