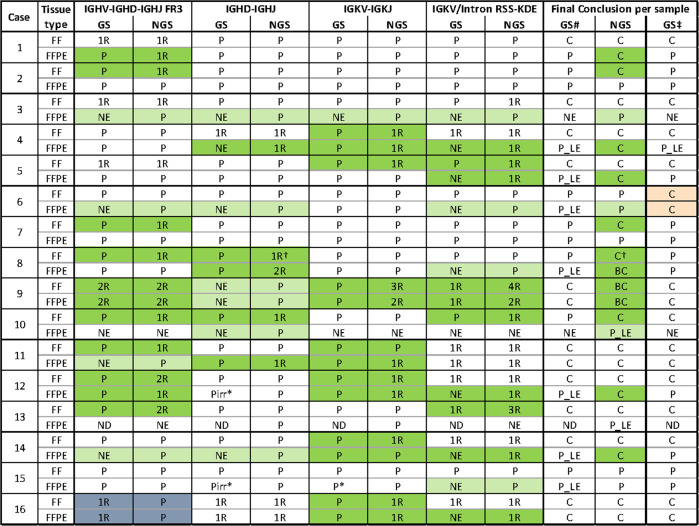# Correction to: Clonality assessment and detection of clonal diversity in classic Hodgkin lymphoma by next-generation sequencing of immunoglobulin gene rearrangements

**DOI:** 10.1038/s41379-021-00986-5

**Published:** 2021-12-15

**Authors:** Diede A. G. van Bladel, Michiel van den Brand, Jos Rijntjes, Samhita Pamidimarri Naga, Demi L. C. M. Haacke, Jeroen A. C. W. Luijks, Konnie M. Hebeda, J. Han J. M. van Krieken, Patricia J. T. A. Groenen, Blanca Scheijen

**Affiliations:** 1 Department of Pathology, Radboud University Medical Center, Nijmegen, The Netherlands; 2 Radboud Institute for Molecular Life Sciences, Nijmegen, The Netherlands; 3 Pathology-DNA, Rijnstate Hospital, Arnhem, The Netherlands; 4 Department of Medical Oncology, Radboud University Medical Center, Nijmegen, The Netherlands

**Keywords:** Immunogenetics, PCR-based techniques

Correction to: *Mod Pathol* 10.1038/s41379-021-00983-8, published online 3 December 2021

The original version of this article unfortunately contained a mistake in the coloring of Table 3. We apologize for the error. The correct table can be found below. The original article has been corrected.