# Supplementary material for: Clonality assessment and detection of clonal diversity in classic Hodgkin lymphoma by next-generation sequencing of immunoglobulin gene rearrangements
Source: Mod Pathol. 2021 Dec 3;35(6):757–66. doi: 10.1038/s41379-021-00983-8 (PMC9174053; doi:10.1038/s41379-021-00983-8)
Supplement: Supplementary file 1 — Supplmentary information. [file 41379_2021_983_MOESM1_ESM.pdf]

Clonality assessment and detection of clonal diversity in classic Hodgkin lymphoma by next-generation sequencing of immunoglobulin gene rearrangements

Diede AG van Bladel, Michiel van den Brand, Jos Rijntjes, Samhita Pamidimarri Naga, Demi LCM Haacke, Jeroen ACW Luijks, Konnie M Hebeda, J Han JM van Krieken, Patricia JTA Groenen, Blanca Scheijen

SUPPLEMENTARY INFORMATION

This file contains Supplementary Materials and Methods, Supplementary Tables, Supplementary Figures and Supplementary Figure Legends. Supplementary Table S4 is an Excel file.

Supplementary Materials and Methods

Immunohistochemistry and in situ hybridization

Histopathological examination was performed on 4 μm tissue sections stained either with hematoxylin-eosin (HE), or with monoclonal antibodies for CD30 (clone BerH2, DAKO, Agilent Technologies Inc., Carpinteria, CA, USA), CD79a (clone JCB117, DAKO), CD2 (clone AB75, Thermo Fisher, Waltham, MA, USA) and CD3 (clone SP7, Thermo Fisher). Sections were stained using EnVision™ FLEX detection system (DAKO) and visualized by EnVision™ FLEX DAB+ Chromogen (DAKO). Epstein-Barr virus (EBV) status of the HRS cells was determined by in situ hybridization for Epstein-Barr virus-encoded small RNAs (EBER) (DAKO). All tissues were scored for EBV status, the presences of HRS cells ($CD30^+$), B cells ($CD79A^+$), T cells ($CD2^+$ or $CD3^+$) and the relative fraction HRS cells to total B cells by at least two independent pathologists (Table 1, Supplementary Table S1).

DNA isolation

Clonality analysis was performed on DNA isolated from fresh frozen (FF) and formalin-fixed paraffin-embedded (FFPE) whole tumor tissues. DNA extraction from FF tissues was performed on five to ten tissue sections of 10µm with TSE (Tris/Saline/EDTA). In short, tissue sections were lysed overnight at 56°C using TSE buffer (10 mM Tris-HCl pH 7.5 / 0.4 M NaCl / 2 mM EDTA pH 8.0) with SDS (20%) and proteinase K (20 mg/ml), followed by ethanol precipitation and DNA was dissolved in Low TE-buffer ($T_{10}E_{0.1}$). For FFPE tissues, Chelex DNA extraction followed by column filtering (QIAamp DNA Micro Kit, Qiagen, Hilden, Germany), or the QIAamp DNA FFPE Tissue Kit (Qiagen) was used to isolate DNA from three to six tissue sections of 10µm. DNA concentrations were determined using Qubit (dsDNA BR Assay Kit, Life Technologies, Carlsbad, CA, USA) and DNA quality was assessed using TapeStation (Genomic DNA ScreenTape Assay, Agilent) analysis according to manufacturer's protocol A DIN score of at least 2.5 is considered sufficient DNA quality for further analyses. For samples with a DIN score

of around 2.0, the success rate of clonality assessment is less and depends on multiple factors like the percentage of neoplastic cells and the location of the DNA double stranded breaks.

Clonality testing by BIOMED-2/EuroClonality assay

Conventional BIOMED-2 clonality testing was performed on FF and FFPE tissue DNA using BIOMED-2/EuroClonality multiplex PCR master mixes for IGH Tube A, B, C and D and IGK Tube A and B (Invivoscribe Inc., San Diego, CA, USA) or custom-ordered reagents, according to the protocol described by the EuroClonality consortium[1]. Analysis of the obtained PCR products was performed by GeneScanning and results were visualized in GeneMarker® V2.6.7 (SoftGenetics, State College, PA, USA). Each sample was analyzed in duplicate for both complete and incomplete rearrangements of IGH (IGHV-IGHD-IGHJ, IGHD-IGHJ) and IGK (IGKV-IGKJ, IGKV-KDE, Intron RSS-KDE). For IGHV-IGHD-IGHJ rearrangements, primers for framework (FR) 1, 2 and 3 were used. The molecular conclusions of all gene rearrangement patterns were scored according to the interpretation guideline of the EuroClonality consortium: 'clonality detected (with polyclonal background)', 'oligoclonality/multiple clones detected', 'polyclonality detected', or 'not evaluable'[2].

Clone identification for cHL cases by IG-NGS

To define threshold values for clone identification in our study that would distinguish a minor cHL clone from a reactive B-cell clone, a series of 30 reactive lymph nodes (study of van den Brand *et al.*[3]) was analyzed for the recurrence of identical dominant clonotypes in duplicate analysis for each of the five targets and compared to distribution in cHL tissue. A clonal rearrangement was defined as a clonotype present in duplicate, with an increased abundancy compared to background B-cell clonotypes. The background was determined as the mean percentage of the 5th, 6th and 7th clonotype, since the 1st to 4th most abundant clonotypes represent the maximum amount of rearrangements present within one clonal B cell, including bi-allelic IGKV-IGKJ and Intron RSS-KDE rearrangements. The normal distribution of gene rearrangements was examined for each of the targets (IGHV-IGHD-IGHJ (FR3), IGHD-IGHJ, IGKV-IGKJ, IGKV-KDE, Intron RSS-KDE) (Supplementary Figure S1). First, the mean percentage of all overlapping clonotypes in a sample was calculated, followed by ranking from the highest to the lowest value (Supplementary Figure S1 B-C). The top-4 overlapping clonotypes were selected for further analysis by calculating the ratio between the percentage of the clonotype and the background, separately for each sample of the duplicate (Supplementary Figure S1 D). Finally, the mean ratio per sample was calculated. The same procedure was performed for the cHL cases and compared to the results of the reactive lymphoproliferative samples to determine the thresholds for clone identification in cHL samples (Supplementary Figure S1 E). For IG gene rearrangements that involved KDE, slightly different approaches were used. The top-4 overlapping clonotypes for IGKV-KDE were selected based

on single-target analysis, after which the ratio's for these clonotypes were calculated based on the combined IGKV-KDE, Intron RSS-KDE analysis. Since Intron RSS-KDE displays only junctional diversity, a different approach was chosen. Here, the two overlapping clonotypes with the highest mean abundancy were selected. The mean percentage of these two clonotypes were used to determine the normal distribution of Intron RSS-KDE rearrangements in reactive lymph node samples. Finally, the values of cHL cases were compared to these results for clone identification. An important note is that there are two Intron RSS-KDE rearrangements, intron -2/0/-3 Kde and intron -0/0/-4 Kde, which are detected with relatively high frequencies in normal B-cell population. A mean percentage that is just above the threshold value for clonality should be interpreted with caution and in the context of clonal rearrangements for other targets.

Enrichment of HRS cells from cHL tissue specimens

HRS cells cases 8 and 16 with representative HRS cell percentages of 3% and 15%, respectively, were enriched by laser microdissection (LMD) for FF tissues and single cell flow cytometric sorting for FFPE tissues. For the isolation of HRS cells by LMD, 9µm thick FF tissue sections were attached to PEN Membrane slides (Leica, Wetzlar, Germany) and stained for CD30 using a standard IHC protocol without antigen retrieval. CD30-positive HRS cells were dissected using a Leica Laser dissection Microscope and collected in lysis buffer for DNA isolation (Supplementary Figure S2 A). Flow cytometry was performed on cell suspensions prepared as described by Juskevicius *et al.*[4]. In short, two FFPE tissue sections of 50µm were prepared, deparaffinized and rehydrated, followed by enzymatic digestion with a cocktail of collagenase type 3, purified collagenase and hyaluronidase (Worthington Biochemical Corp., Lakewood, NJ, USA). Cell suspensions were fluorescently labeled for CD30 (CD30 primary antibody, clone BerH2, DAKO, 1:50 ; Alexa Fluor 647 conjugated secondary antibody α-mouse IgG F(ab) fragment, Thermo Fisher, 1:2000), IRF4/MUM1 (MUM1 primary antibody, clone EPR5653, Abcam, Cambridge, UK, 1:100 ; biotinylated secondary antibody α-rabbit IgG, Vector Laboratories, Burlingame, CA, USA, 1:200 ; Streptavidin PE, Invitrogen, Carlsbad, CA, USA, 1:400) and 4', 6 diamidino-2-phenylindole (DAPI, 1 mg/mL, Sigma-Aldrich). After selection of $DAPI^+$ cells, $CD30^+MUM1^+$ double positive HRS cells were sorted on a FACS Aria (BD Biosciences, San Jose, CA, USA). Gating strategy to sort the HRS cell population is shown in Supplementary Figure S2 B. DNA extraction from the enriched HRS cells (both FF and FFPE tissues) was performed using the Arcturus PicoPure DNA Extraction Kit (Thermo Fisher). Subsequently, the HRS cell enriched DNA samples were analyzed with IG-NGS using 40-50 ng input DNA for flow-sorted cells and 0.5-0.8 ng DNA for LMD samples.

Single-cell analysis on DEPArray-sorted HRS cells

DNA integrity of the FFPE specimens was assessed using the DEPArray™ FFPE QC Kit (Menarini Silicon Biosystems, Bologna, Italy). The DNA quality was assessed by the difference in quantification cycles (ΔCq) obtained with the long amplicon assay (132 bp) and the short amplicon assay (54 bp) for a given sample using the formula: $QC\ score = \frac{1}{2^{\Delta \mathrm{Cq}}}$. Samples with a QC value of at least 0.3 are of sufficient quality for further analysis. For case 9, 21 single HRS cells, 9 rosetted HRS cells and a leukocyte pool (10 cells) were processed for analysis after isolation from FFPE tissue (two sections of 50 µm) with DEPArray™ sorting technology (Menarini Silicon Biosystems), based on CD30 and PD-L1 expression. Following HRS cell isolation, genomic DNA was isolated, amplified with Ampli1™ Whole Genome Amplification kit, and DNA libraries were prepared using the Ampli1™ LowPass kit (both Menarini Silicon Biosystems) for sequencing on Illumina MiSeq, as described previously[5]. Copy Number Variation (CNV) profiles were generated using the CNV caller ichorCNA[6] with default settings. For samples of male patients, this tool re-scales the counts of each bin in Chromosome X with the median counts of this chromosome. Further, unsupervised hierarchical clustering was performed using the R package heatmap3 to analyze the CNV profiles.

Supplementary Figures and Tables

Supplementary Table S1.

Characteristics of classic Hodgkin lymphoma fresh frozen tissue specimens.

Case	Diagnosis	Gender	Age at diagnosis (y)	% HRS cells	% Total B cells	% HRS cells of total B cells
1	NSHL	M	14	ND	ND	ND
2	NSHL	M	14	10	40	20
3	MCHL	F	14	2	40	5
4	NSHL	F	15	10	10	50
5	NSHL	F	17	5	60	8
6	NSHL	M	22	15	20	43
7	MCHL	M	22	1	50	2
8	NSHL	M	33	3	20	13
9	NSHL	M	38	5	15	25
10	MCHL	M	47	5	40	11
11	NSHL	M	76	5	15	25
12	NSHL	F	19	5	10	33
13	MCHL	M	44	2	10	17
14	MCHL	M	32	20	20	50
15	NSHL	M	61	2	35	5
16	NSHL	M	67	10	40	20

The fraction of HRS cells and background B cells based on HE and CD30 (% HRS cells) and CD79a (B cells) on fresh frozen tissue sections. Cell percentages may differ from corresponding FFPE specimen, since different parts of the tumor were used for processing into FF and FFPE tissue blocks and the subjectivity of scoring.

Supplementary Table S2.

Characteristics of reactive lymphoproliferative samples.

	van den Brand *et al.* 2021[3]	Diagnosis	Location	Source
**RLN-1**	EC-BV-24	Reactive	Thymus	FFPE
**RLN-2**	EC-BV-25	Reactive	Lymph node, inguinal	FFPE
**RLN-3**	EC-BV-28	Reactive	Lymph node, submental	FFPE
**RLN-4**	EC-BV-30	Reactive, NOS	Lymph node, mediastinum	FFPE
**RLN-5**	EC-BV-32	Reactive, NOS	Lymph node, cervical	FFPE
**RLN-6**	EC-BV-34	Reactive, NOS	Nasopharynx	FFPE
**RLN-7**	EC-BV-35	Reactive, NOS	Lymph node, cervical	FFPE
**RLN-8**	EC-BV-41	Reactive, NOS	Lymph node, cervical	FFPE
**RLN-9**	EC-BV-47	Reactive, NOS	Lung	FFPE
**RLN-10**	EC-BV-52	Reactive, NOS	Skin	FFPE
**RLN-11**	EC-BV-53	Reactive, NOS	Lymph node, retroperitoneum	FFPE
**RLN-12**	EC-BV-58	Reactive LN	Lymph node, cervical	FFPE
**RLN-13**	EC-BV-62	Reactive LN	Lymph node, inguinal	FFPE
**RLN-14**	EC-BV-93	Reactive, NOS	UK	FFPE
**RLN-15**	EC-BV-96	Reactive, NOS	UK	FFPE
**RLN-16**	NL8	Reactive tonsil	Tonsil	FF
**RLN-17**	GBS12	Reactive tonsil	Tonsil	FF
**RLN-18**	GBS13	Reactive tonsil	Tonsil	FF
**RLN-19**	GBN7	Reactive LN	UK	FF
**RLN-20**	EC-BV-116	Reactive tonsil	Tonsil	FFPE
**RLN-21**	EC-BV-118	Reactive tonsil	Tonsil	FFPE
**RLN-22**	EC-BV-128	Reactive LN	UK	FFPE
**RLN-23**	EC-BV-132	Reactive tonsil	Tonsil	FFPE
**RLN-24**	EC-BV-135	Reactive tonsil	Tonsil	FFPE
**RLN-25**	EC-BV-136	Reactive tonsil	Tonsil	FFPE
**RLN-26**	EC-BV-138	Reactive tonsil	Tonsil	FFPE
**RLN-27**	EC-BV-141	Reactive tonsil	Tonsil	FFPE
**RLN-28**	EC-BV-144	Reactive tonsil	Tonsil	FFPE
**RLN-29**	EC-BV-147	Reactive tonsil	Tonsil	FFPE
**RLN-30**	EC-BV-148	Reactive tonsil	Tonsil	FFPE

NOS: Not otherwise specified; LN: Lymph node; UK: Unknown.

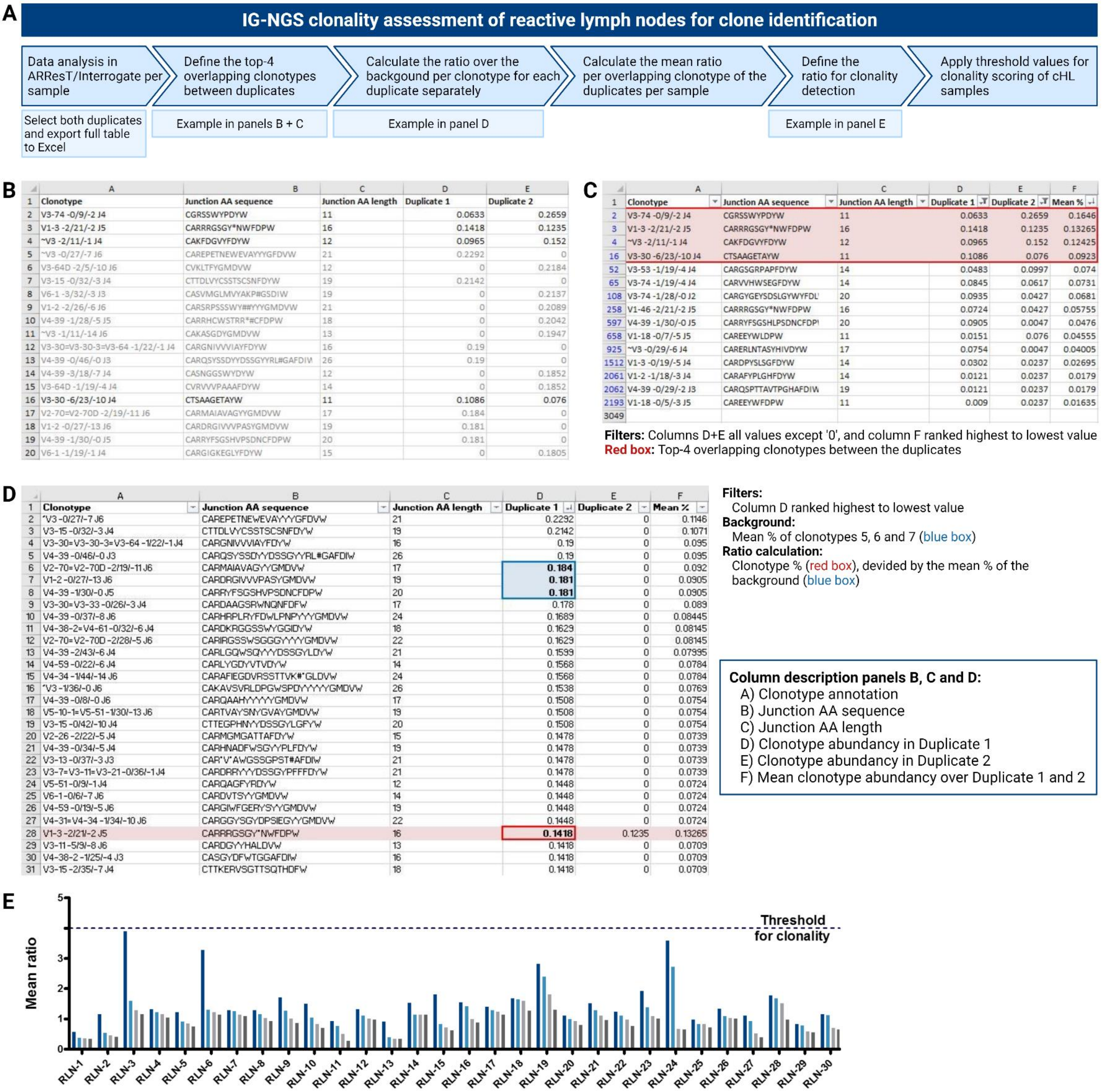

B

	A	B	C	D	E
1	Clonotype	Junction AA sequence	Junction AA length	Duplicate 1	Duplicate 2
2	V3-74 -0/9/-2 J4	CGRSSWYPDYW	11	0.0633	0.2659
3	V1-3 -2/21/-2 J5	CARRRGSGY*NWFDPW	16	0.1418	0.1235
4	~V3 -2/11/-1 J4	CAKFDGVYFDYW	12	0.0965	0.152
5	~V3 -0/27/-7 J6	CAREPETNEWEVAYYYGFDVW	21	0.2292	0
6	V3-64D -2/5/-10 J6	CVKLTFYGMDVW	12	0	0.2184
7	V3-15 -0/32/-3 J4	CTTDLVYCSSTSCSNFDYW	19	0.2142	0
8	V6-1 -3/32/-3 J3	CASVMGLMVYAKP#GSDIW	19	0	0.2137
9	V1-2 -2/26/-6 J6	CARSRPSSSWY##YYYGMDVW	21	0	0.2089
10	V4-39 -1/28/-5 J5	CARRHCWSTRR*#CFDPW	18	0	0.2042
11	~V3 -1/11/-14 J6	CAKASGDYGMDVW	13	0	0.1947
12	V3-30=V3-30-3=V3-64 -1/22/-1 J4	CARGNIVVVIAYFDYW	16	0.19	0
13	V4-39 -0/46/-0 J3	CARQSYSSDYYDSSGYYRL#GAFDIW	26	0.19	0
14	V4-39 -3/18/-7 J4	CASNGGSWYDYW	12	0	0.1852
15	V3-64D -1/19/-4 J4	CVRVVVPAAAFDYW	14	0	0.1852
16	V3-30 -6/23/-10 J4	CTSAAGETAYW	11	0.1086	0.076
17	V2-70=V2-70D -2/19/-11 J6	CARMAIAVAGYYGMDVW	17	0.184	0
18	V1-2 -0/27/-13 J6	CARDRGIVVVPASYGMDVW	19	0.181	0
19	V4-39 -1/30/-0 J5	CARRYFSGSHVPSDNCFDPW	20	0.181	0
20	V6-1 -1/19/-1 J4	CARGIGKEGLYFDYW	15	0	0.1805

C

	A		C	D	E	F
1	Clonotype	Junction AA sequence	Junction AA length	Duplicate 1	Duplicate 2	Mean %
2	V3-74 -0/9/-2 J4	CGRSSWYPDYW	11	0.0633	0.2659	0.1646
3	V1-3 -2/21/-2 J5	CARRRGSGY*NWFDPW	16	0.1418	0.1235	0.13265
4	~V3 -2/11/-1 J4	CAKFDGVYFDYW	12	0.0965	0.152	0.12425
16	V3-30 -6/23/-10 J4	CTSAAGETAYW	11	0.1086	0.076	0.0923
52	V3-53 -1/19/-4 J4	CARGSGRPAPFDYW	14	0.0483	0.0997	0.074
65	V3-74 -1/19/-4 J4	CARVVHWSEGFDYW	14	0.0845	0.0617	0.0731
108	V3-74 -1/28/-0 J2	CARGYGEYSDSLGYWYFDL'	20	0.0935	0.0427	0.0681
258	V1-46 -2/21/-2 J5	CARRRGSGY*NWFDPW	16	0.0724	0.0427	0.05755
597	V4-39 -1/30/-0 J5	CARRYFSGSHLPSDNCFDP'	20	0.0905	0.0047	0.0476
658	V1-18 -0/7/-5 J5	CAREEYWLDPW	11	0.0151	0.076	0.04555
925	~V3 -0/29/-6 J4	CARERLNTASYHIVDYW	17	0.0754	0.0047	0.04005
1512	V1-3 -0/19/-5 J4	CARDPYSLSGFDYW	14	0.0302	0.0237	0.02695
2061	V1-2 -1/18/-3 J4	CARAFYPLGHFDYW	14	0.0121	0.0237	0.0179
2062	V4-39 -0/29/-2 J3	CARQSPTTAVTPGHAFDIW	19	0.0121	0.0237	0.0179
2193	V1-18 -0/5/-3 J5	CAREEYWFDPW	11	0.009	0.0237	0.01635
3049						

D

	A	B	C	D	E	F
1	Clonotype	Junction AA sequence	Junction AA length	Duplicate 1	Duplicate 2	Mean %
2	~V3 -0/27/-7 J6	CAREPETNEWEVAYYYGFDVW	21	0.2292	0	0.1146
3	V3-15 -0/32/-3 J4	CTTDLVYCSSTSCSNFDYW	19	0.2142	0	0.1071
4	V3-30=V3-30-3=V3-64 -1/22/-1 J4	CARGNIVVVIAYFDYW	16	0.19	0	0.095
5	V4-39 -0/46/-0 J3	CARQSYSSDYYDSSGYYRL#GAFDIW	26	0.19	0	0.095
6	V2-70=V2-70D -2/19/-11 J6	CARMAIAVAGYYGMDVW	17	**0.184**	0	0.092
7	V1-2 -0/27/-13 J6	CARDRGIVVVPASYGMDVW	19	**0.181**	0	0.0905
8	V4-39 -1/30/-0 J5	CARRYFSGSHVPSDNCFDPW	20	**0.181**	0	0.0905
9	V3-30=V3-33 -0/26/-3 J4	CARDAAGSRWNQNFDFW	17	0.178	0	0.089
10	V4-39 -0/37/-8 J6	CARHRPLRYFDWLPNPYYYGMDVW	24	0.1689	0	0.08445
11	V4-38-2=V4-61 -0/32/-6 J4	CARDKRGGSSWYGGIDYW	18	0.1629	0	0.08145
12	V2-70=V2-70D -2/28/-5 J6	CARIRGSSWSGGGYYYYGMDVW	22	0.1629	0	0.08145
13	V4-39 -2/43/-6 J4	CARLGQWSQYYYDSSGYLDYW	21	0.1599	0	0.07995
14	V4-59 -0/22/-6 J4	CARLYGDYVTVDYW	14	0.1568	0	0.0784
15	V4-34 -1/44/-14 J6	CARAFIEGDVRSSTTVK#*GLDVW	24	0.1568	0	0.0784
16	~V3 -1/36/-0 J6	CAKAVSVRLDPGWSPDYYYYYGMDVW	26	0.1538	0	0.0769
17	V4-39 -0/8/-0 J6	CARQAAHYYYYYGMDVW	17	0.1508	0	0.0754
18	V5-10-1=V5-51 -1/30/-13 J6	CARTVAYSNYGVAYGMDVW	19	0.1508	0	0.0754
19	V3-15 -0/42/-10 J4	CTTEGPHNYYDSSGYLGFYW	20	0.1508	0	0.0754
20	V2-26 -2/22/-5 J4	CARMGMGATTAFDYW	15	0.1478	0	0.0739
21	V4-39 -0/34/-5 J4	CARHNADFWSGYYPLFDYW	19	0.1478	0	0.0739
22	V3-13 -0/37/-3 J3	CAR*V*AWGSSGPST#AFDIW	21	0.1478	0	0.0739
23	V3-7=V3-11=V3-21 -0/36/-1 J4	CARDRRYYYDSSGYPFFFDYW	21	0.1478	0	0.0739
24	V5-51 -0/9/-1 J4	CARQAGFYRDYW	12	0.1448	0	0.0724
25	V6-1 -0/6/-7 J6	CARDVTSYYGMDVW	14	0.1448	0	0.0724
26	V4-59 -0/19/-5 J6	CARGIWFGERYSYYGMDVW	19	0.1448	0	0.0724
27	V4-31=V4-34 -1/34/-10 J6	CARGGYSGYDPSIEGYYGMDVW	22	0.1448	0	0.0724
28	V1-3 -2/21/-2 J5	CARRRGSGY*NWFDPW	16	**0.1418**	0.1235	0.13265
29	V3-11 -5/9/-8 J6	CARDGYYHALDVW	13	0.1418	0	0.0709
30	V4-38-2 -1/25/-4 J3	CASGYDFWTGGAFDIW	16	0.1418	0	0.0709
31	V3-15 -2/35/-7 J4	CTTKERVSGTTSQTHDFW	18	0.1418	0	0.0709

Supplementary Figure S1. Outline duplicate analysis of reactive lymphoproliferative samples for clone identification. All steps of the data analysis are shown as flow scheme in panel A. Screenshots of the Excel output from ARResT/Interrogate for IGHV-IGHD-IGHJ (FR3) of RLN-7 are shown as an example. Each line represents a single clonotype with a unique nucleotide sequence translated as a junctional amino acid sequence. (B) Clonotypes that are present in both duplicates (overlapping clonotypes) are shown in black, non-overlapping clonotypes are shown in grey. (C) Data is filtered to keep only overlapping clonotypes. For each clonotypes, the mean % (column F) is calculated over columns D and E and ranked from the highest to the lowest value. The top-4 overlapping clonotypes (red box) are selected for further calculations. (D) All filters are removed and the clonotypes are ranked from the highest to the lowest percentage within Duplicate 1 (column D). The background is defined as the mean % of clonotypes 5, 6 and 7 (shown in blue). For each of the 4 selected overlapping clonotypes (in red), the ratio compared to the background is calculated. This step is repeated for Duplicate 2 (column E) to complete the results of this sample for IGHV-IGHD-IGHJ (FR3). The complete procedure is repeated for all other samples and for each target. (E) Once all samples are analyzed for a single target, the ratio value for clonality testing can be defined by setting the threshold just above the highest ratio found in these reactive lymphoproliferative samples.

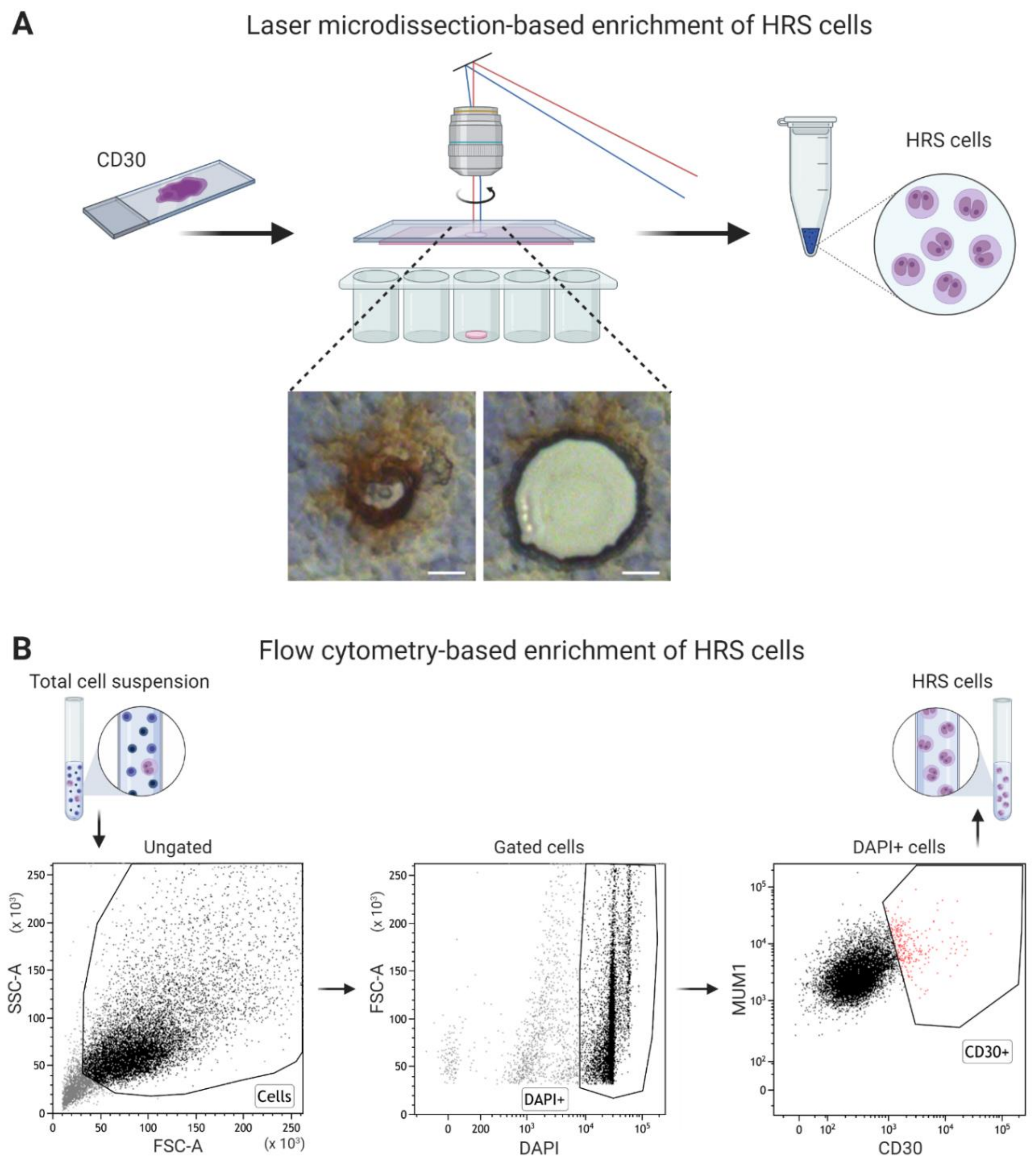

Supplementary Figure S2. Enrichment of HRS cells from classic Hodgkin lymphoma tumor tissue. (A) Schematic overview of the workflow for HRS cell enrichment of fresh frozen (FF) tumor tissue using laser microdissection (LMD). Scale bars indicate 10µm. (B) FFPE tumor tissue was enriched using flow cytometry after mild enzymatic digestion of FFPE specimens, followed by DAPI, anti-MUM1 and anti-CD30 staining. Gating strategy for sorting the HRS cells is shown. First a gate was set to remove cell debris, then $DAPI^{+}$ cells were selected, followed by gating both $MUM1^{+}CD30^{+}$ and $CD30^{-}$ cells. $MUM1^{+}CD30^{+}$ cells (red events) were sorted as HRS cell fraction.

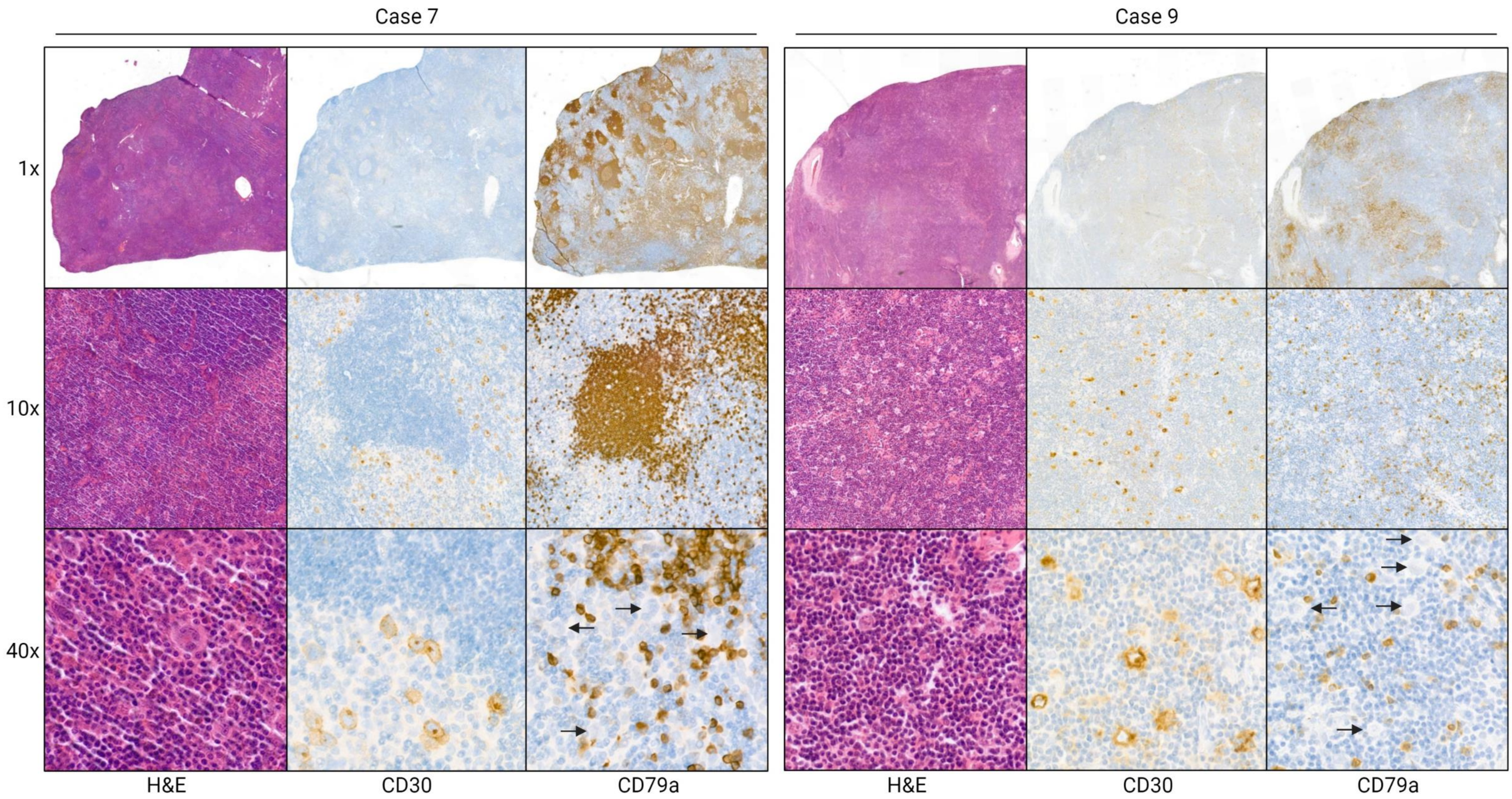

Supplementary Figure S3. Immunohistochemical staining of cHL tumor tissues. Tissue sections are stained for H&E, CD30 and CD79a and shown as 1x, 10x and 40x magnifications for case 7 (left panel) and case 9 (right panel). Hodgkin Reed-Sternberg (HRS) cells are present as CD30 positive/CD79a negative (DAB staining; black arrows indicate CD79a negative HRS cells), large bi- or multinucleated cells.

Supplementary Table S3.

Overview results of BIOMED-2/Genescan analysis for each classic Hodgkin lymphoma sample.

			**BIOMED-2/Genescan**					**IG-NGS**					
**Case**	**Tissue Type**	**DIN score**	**IGHV-IGHD-IGHJ (FR3)**	**IGHD-IGHJ**	**IGKV-IGKJ**	**IGKV/ Intron RSS-KDE**	**Conclusion**	**IGHV-IGHD-IGHJ (FR3)**	**IGHD-IGHJ**	**IGKV-IGKJ**	**IGKV-KDE**	**Intron RSS-KDE**	**Conclusion**
**1**	**FF**	2.3	**C + PCB**	P	P	P	**Clonal**	**C + PCB**	P	P	P	P	**Clonal**
	**FFPE**	3.2	P	P	P	P	Polyclonal	**C + PCB**	P	P	P	P	**Clonal**
**2**	**FF**	8.2	P	P	P	P	Polyclonal	**C + PCB**	P	P	P	P	**Clonal**
	**FFPE**	5.2	P	P	P	P	Polyclonal	P	P	P	P	P	Polyclonal
**3**	**FF**	7.5	**C + PCB***	P	P	P	**Clonal**	**C + PCB**	P	P	P	**C + PCB**	**Clonal**
	**FFPE**	1.9	NE	NE	NE	NE	Not evaluable	Pirr	P	P	Pirr	P	Polyclonal
**4**	**FF**	9.2	P*	**C + PCB**	P	**C + PCB**	**Clonal**	P	**C + PCB**	**C + PCB**	P	**C + PCB**	**Clonal**
	**FFPE**	2.2	P	NE	P	NE	Polyclonal_LE	P	**C + PCB**	**C + PCB**	P	**C + PCB**	**Clonal**
**5**	**FF**	8.1	**C + PCB***	P	P	P	**Clonal**	**C + PCB**	P	**C + PCB**	P	**C + PCB**	**Clonal**
	**FFPE**	3.2	**P**	P	P	NE	Polyclonal_LE	P	P	P	P	**C + PCB**	**Clonal**
**6**	**FF**	5.6	P*	P	P	P	Polyclonal	P	P	P	P	P	Polyclonal
	**FFPE**	8.0	NE*	NE	P	NE	Polyclonal_LE	P	P	P	P	P	Polyclonal
**7**	**FF**	3.0	P	P	P	P	Polyclonal	**C + PCB**	P	P	P	P	**Clonal**
	**FFPE**	4.5	P	P	P	P	Polyclonal	P	P	P	P	P	Polyclonal
**8**	**FF**	7.9	P	P	P	P	Polyclonal	**C + PCB**	**C + PCB‡**	P	P	P	**Clonal‡**
	**FFPE**	4.0	P	P	P	NE	Polyclonal_LE	P	**C/C + PCB**	P	P	P	**Biclonal**
**9**	**FF**	3.9	**C/C + PCB***	NE	P	**C + PCB**	**Clonal**	**C/C + PCB**	P	**C/C/C + PCB**	**C/C/C + PCB**	**C/C/C + PCB**	**Biclonal**
	**FFPE**	3.3	**C/C + PCB**	NE	P	**C + PCB**	**Clonal**	**C/C + PCB**	P	**C/C + PCB**	P	**C/C + PCB**	**Biclonal**
**10**	**FF**	8.1	P*	P	P	P	Polyclonal	**C + PCB**	**C + PCB**	P	P	C + PCB	**Clonal**
	**FFPE**	2.1	NE	NE	NE	NE	Not evaluable	NE	Pirr	NE	NE	NE	Polyclonal_LE
**11**	**FF**	8.2	P	P	P	**C + PCB**	**Clonal**	**C + PCB**	P	P	**C + PCB**	P	**Clonal**
	**FFPE**	3.3	NE	P	P	**C + PCB**	**Clonal**	P	**C + PCB**	**C + PCB**	**C + PCB**	P	**Clonal**
**12**	**FF**	6.2	P*	P	P	**C + PCB**	**Clonal**	**C/C + PCB**	P	**C + PCB**	**C + PCB**	P	**Clonal**
	**FFPE**	2.9	P	Pirr#	P	NE	Polyclonal_LE	**C + PCB**	P	**C + PCB**	**C + PCB**	P	**Clonal**
**13**	**FF**	8.0	P	P	P	**C + PCB**	**Clonal**	**C/C + PCB**	P	P	**C/C + PCB**	**C + PCB**	**Clonal**
	**FFPE**	1.8	ND	ND	ND	ND	ND	NE	Pirr	Pirr	NE	NE	Polyclonal_LE
**14**	**FF**	6.9	P	P	P	**C + PCB**	**Clonal**	P	P	**C + PCB**	P	**C + PCB**	**Clonal**
	**FFPE**	2.7	NE	NE	P	NE	Polyclonal_LE	P	P	P	P	**C + PCB**	**Clonal**
**15**	**FF**	7.2	P	P	P	P	Polyclonal	P	P	P	P	P	Polyclonal
	**FFPE**	1.7	P	Pirr#	P#	NE	Polyclonal_LE	P	P	P	P	P	Polyclonal
**16**	**FF**	6.5	**C + PCB**	**C + PCB**	P	**C + PCB**	**Clonal**	P	**C + PCB**	**C + PCB**	P	**C + PCB**	**Clonal**
	**FFPE**	3.1	**C + PCB**	**C + PCB**	P	**C + PCB**	**Clonal**	P	**C + PCB**	**C + PCB**	P	**C + PCB**	**Clonal**

Results are shown for both FF and FFPE tissue sections for conventional (left panel) and IG-NGS clonality testing (right panel). Rearrangements involving KDE are scored combined for BIOMED-2/EuroClonality assay, according to standardized evaluation, while for IG-NGS, the IGKV-IGKDE and intron-IGKDE are evaluated separately in this study. DNA quality was assessed using TapeStation (Genomic DNA ScreenTape Assay), of which the DIN score is shown as quality indicator. C + PCB: Clonal with polyclonal background; P: Polyclonal; Pirr: Polyclonal irregular pattern; NE: not evaluable; ND: not done; Polyclonal_LE: polyclonal_less evaluable, results are based on less than 4 interpretable IG targets and therefore potential clonal results can be missed, resulting in a molecular conclusion that may be less reliable compared to samples with interpretable results on all 4 IG targets. Number of C's represents the number of clonal rearrangements. * Clonal rearrangements are detected using FR1 and/or FR2 primers; # IGHD1/2/3/5-IGHJ and IGKV2/4/5 are not evaluable, due to suboptimal DNA quality; ‡ The second clonal IGHD-IGHJ rearrangement was observed in HRS enriched FF DNA fraction (see Supplemental Table S5).

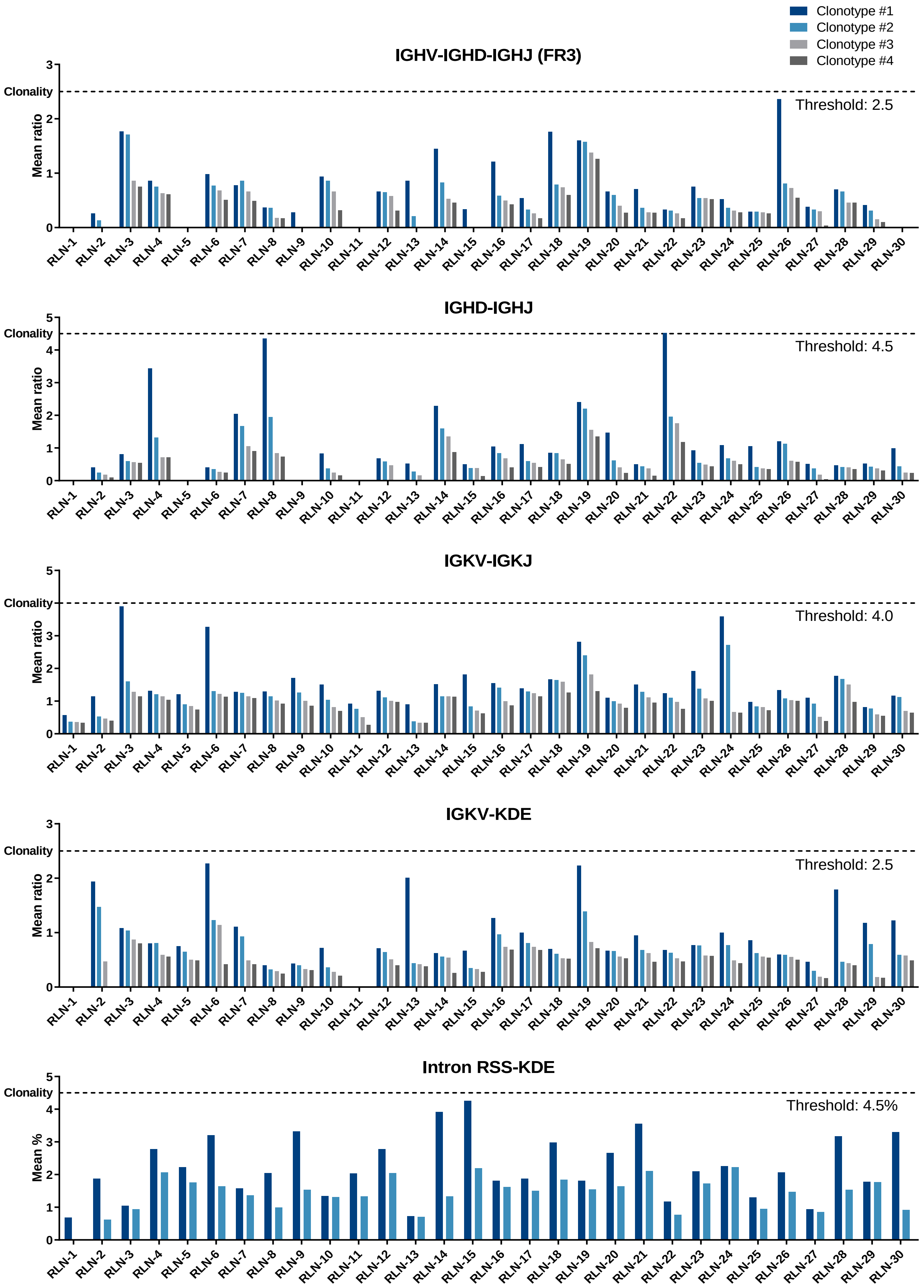

Supplemental Figure S4. Distribution of IG gene rearrangements in reactive lymphoproliferative samples. Mean ratios of the top 4 the most abundant clonotypes are shown for 30 reactive lymphoproliferative samples, for IGHV-IGHD-IGHJ (FR3), IGHD-IGHJ, IGKV-IGKJ and IGKV-KDE. For Intron RSS-KDE rearrangements, the mean percentage of the two most abundantly present clonotypes are shown.

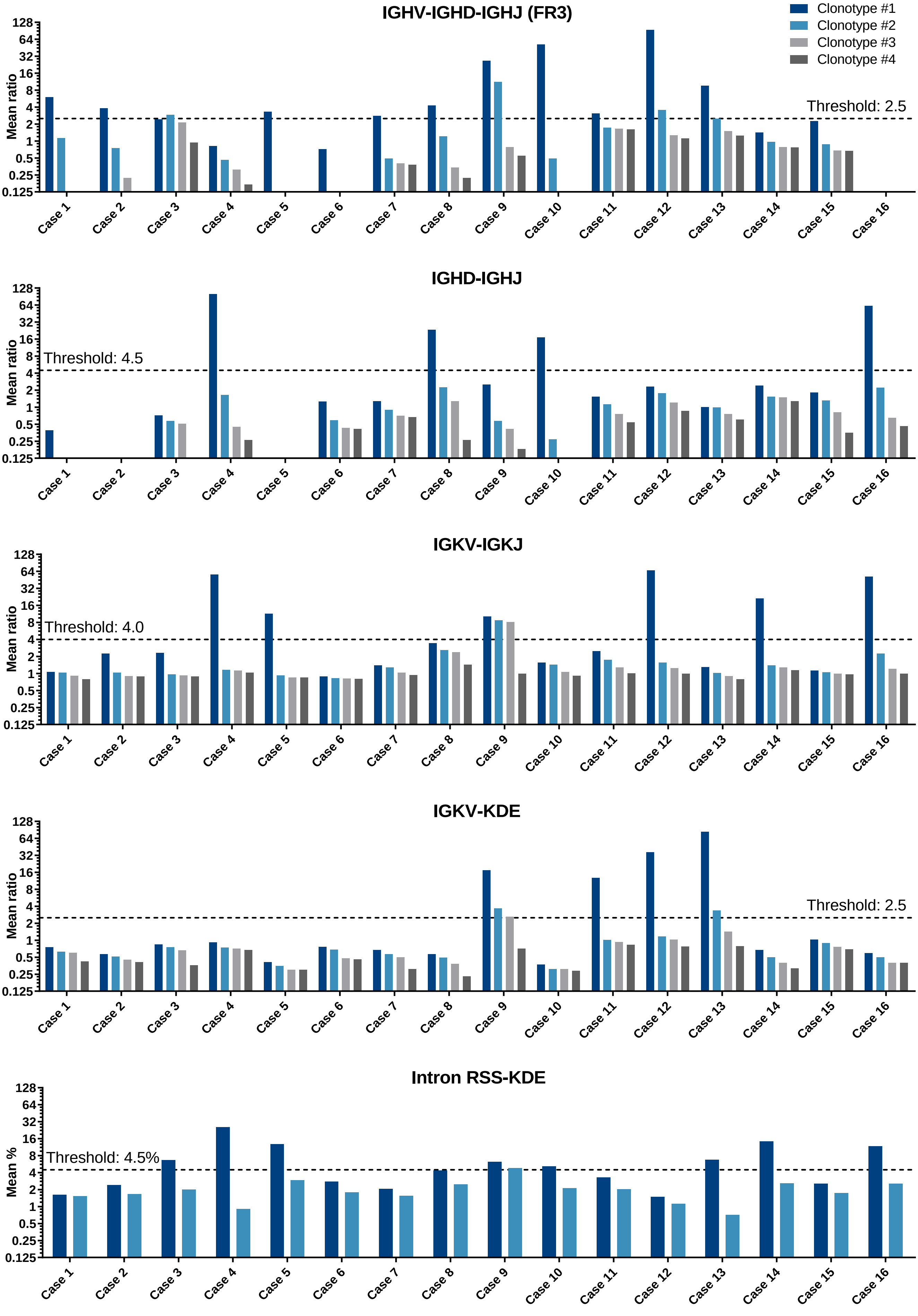

Supplementary Figure S5. Detection of clonal IG gene rearrangements in classic Hodgkin lymphoma on the basis of thresholds determined in lymphoproliferative tissues. Clonal rearrangements are defined for fresh frozen cHL tumor tissue, based on the distribution of rearrangements in reactive lymphoproliferative samples. Mean ratios of the 4 most abundantly present clonotypes are shown for IGHV-IGHD-IGHJ, IGHD-IGHJ, IGKV-IGKJ and IGKV-KDE. For Intron RSS-KDE rearrangements, the mean percentage of the two most abundantly present clonotypes are shown.

IG Heavy chain

```
IGH V3-48=V369-1                                                        Mismatches (%)
Germline       CTGAGAGCCGAGGACACGGCTGTTTATTACTGTGCGAGAGA
               ||||||||||||||||||||| ||||||||||| |||||||                2/41 (4.9%)
Case 1         CTGAGAGCCGAGGACACGGCTATTTATTACTGTACGAGAGA

IGH V1-3
Germline       CTGAGATCTGAAGACACGGCTGTGTATTACTGTGCGAGAGA
               || | |||||||||||||||| | ||||| |||||||||||                5/41 (12.2%)
Case 2         CTCACATCTGAAGACACGGCTATCTATTATTGTGCGAGAGA

IGH V3
Germline       CTGAGAGCCGAGGACACGGCCGTATATTACTGTGCGA
               ||||||| ||| ||| |||| |||||||| |||||||                    5/37 (13.5%)
Case 7         CTGAGAGGCGACGACTCGGCTGTATATTATTGTGCGA

IGH V3-9
Germline       CTGAGAGCTGAGGACACGGCCTTGTATTACTGTGCAAAAGAT
               ||||||| || |||||||||| ||||||||||||||||||||               3/42 (7.1%)
Case 8         CTGAGAGGTGCGGACACGGCCCTGTATTACTGTGCAAAAGAT

IGH V3-71
Germline       CTGAGAGCCGAGGACACGGCCGTGTATTACTGTGC
               || |||| ||| ||||| |||||||||||||||||                      4/35 (11.4%)
Case 10        CTTAGAGGCGACGACACAGCCGTGTATTACTGTGC

IGH V3-9
Germline       CTGAGAGCTGAGGACACGGCCTTGTATTACTGTGCAAAAGA
               ||||  ||||||||||||||| ||||||| ||||  |||||                6/41 (14.6%)
Case 12        CTGAAGGCTGAGGACACGGCCCTGTATTATTGTGTTAAAGA

IGH V3-74
Germline       CTGAGAGCCGAGGACACGGCTGTGTATTACTGTGCAAG
                |||| | ||||||||||||||||||||||||| ||||                   4/38 (10.5%)
Case 13        GTGAGGGTCGAGGACACGGCTGTGTATTACTGTACAAG
```

IG Kappa light chain

```
IGK V1-33=V1D-33
Germline       CTGCAGCCTGAAGATATTGCAACATATTACTGTCAACAGTATGATAA
               ||||||   |||||| |||||||||||||||||||||||| |||| |          6/47 (12.8%)
Case 12        CTGCAGAGGGAAGATTTTGCAACATATTACTGTCAACAGTCTGATGA

IGK V4-1
Germline       CTGCAGGCTGAAGATGTGGCAGTTTATTACTGTCAGCAATATTATAGTACTCCTC
               |||||||||||||||||||||||||||||||||||||| ||||||||||||||||  1/55 (1.8%)
Case 16        CTGCAGGCTGAAGATGTGGCAGTTTATTACTGTCAGCAGTATTATAGTACTCCTC
```

Supplementary Figure S6. Somatic mutations in IGHV and IGKV genes of dominant clonal gene rearrangements. The obtained nucleotide sequences of representative cases (FF) are aligned with the reference sequence (Germline) of the corresponding IGHV (upper panel) or IGKV (lower panel) genes. The number (and percentage) of mismatches is shown for the overlapping sequence. Due to the relatively short amplicons, there is only a small part of the IGHV and IGKV gene sequence obtained from the dominant clonal gene rearrangements. Nucleotide substitutions are found in replicate analysis (independent NGS data sets) for both FF and FFPE.

Supplementary Table S5.

NGS-based clonality assessment in HRS-enriched samples. Enrichment of HRS cells with LMD was performed on FF tissue, flow sort enrichment on FFPE tissue and results are compared to whole tumor tissue of the same origin. Clonotypes are defined by the 5’ and 3’ gene annotation and the nucleotide sequence of the junction of the rearrangement. Due to limited DNA yields after LMD enrichment, the analysis was only performed for the IGHD-IGHJ target. Shown percentages are the mean values of duplicate results, except for LMD enrichments (both cases) and IGHV-IGHD-IGHJ result for HRS cell fraction of case 8.

Case	Sample	IGHV-IGHD-IGHJ		IGHD-IGHJ		IGKV-IGKJ		IGKV/Intron RSS-KDE	
		Clonotype	%	Clonotype	%	Clonotype	%	Clonotype	%
8	**Whole tumor tissue FF**	V3-9 -1/20/-6 J3	1.3	D3-9 -3/33/-5 J6<br>D1-26 -2/8/-3 J5	10.8<br>1.0	P	-	P	-
	**LMD enriched HRS cells***			D3-9 -3/33/-5 J6<br>D1-26 -2/8/-3 J5	40.4<br>39.3				
	**Whole tumor tissue FFPE**	P	-	D3-9 -3/33/-5 J6<br>D1-26 -2/8/-3 J5	1.5<br>2.6	P	-	P	-
	**HRS cell fraction**	V3-9 -1/20/-6 J3	69.7	D3-9 -3/33/-5 J6<br>D1-26 -2/8/-3 J5	33.4<br>13.8	P	-	P	-
16	**Whole tumor tissue FF**	P	-	D6-6 -0/5/-5 J5	14.5	V4-1 -1/0/-1 J2	15.5	intron -6/6/-11 Kde	11.8
	**LMD enriched HRS cells***			D6-6 -0/5/-5 J5	49.5				
	**Whole tumor tissue FFPE**	P	-	D6-6 -0/5/-5 J5	22.9	V4-1 -1/0/-1 J2	23.4	intron -6/6/-11 Kde	20.6
	**HRS cell fraction**	P	-	D6-6 -0/5/-5 J5	80.7	V4-1 -1/0/-1 J2	78.4	intron -6/6/-11 Kde	72.1

***** Due to limited DNA yields after LMD enrichment, only the IGH-DJ target is analyzed.

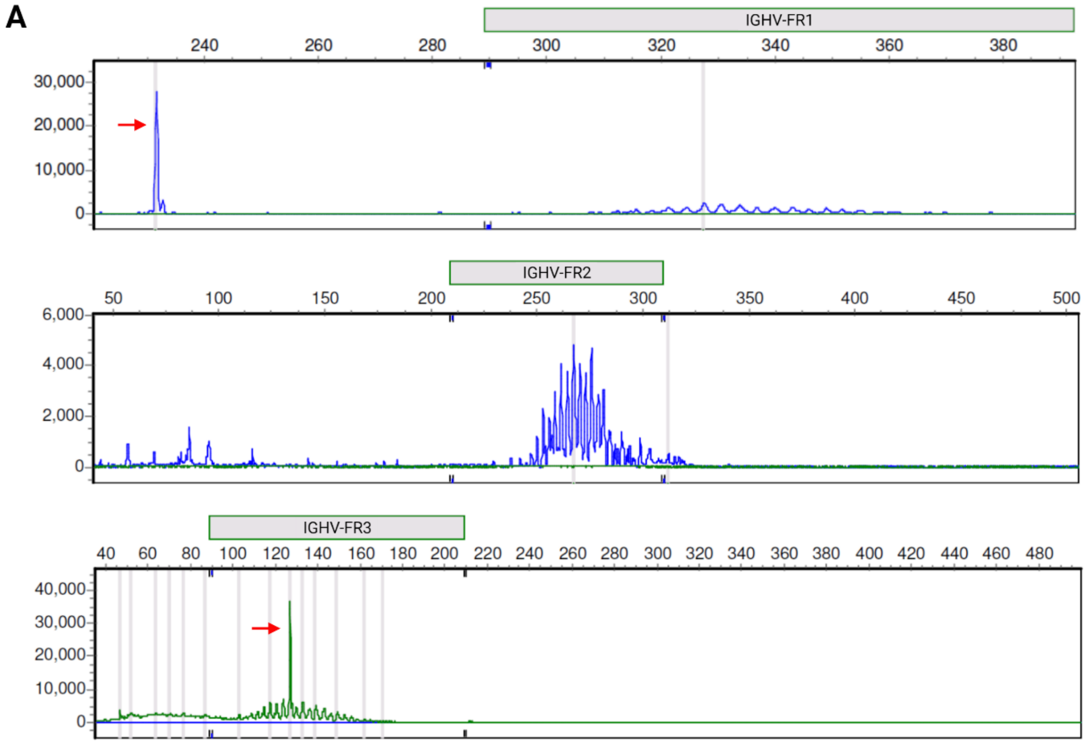

B Germline sequence IGHV3-74

GACTGCACACAGAGAACTCACCATGGAGTTTGGGCTGAGCTGGGTTTTCCTTGTTGCTATTTTAAAAGGTGATTCATG
GAGAACTGGAGATATGGAGTGTGAATGGACATGAGTGAGATAAGCAGTGGATGTGTGTGGCAGTTTCTGACCAGGGTG
TCTCTGTGTTTGCAGGTGTCCAGTGTGAGGTGCAGCTGGTGGAGTCCGGGGGAGGCTTAGTTCAGCCTGGGGGGTCCC
TGAGACTCTCCTGTGCAGCCTCTGGATTCACCTTCAGTAGCTACTGGATGCACTGGGTCCGCCAAGCtccagggaagg
ggctggtgtgggtctcacgtattaatagtgatgggagtagcacaagctacgcggactccgtgaagggccgattcacca
tctccagaGACAACGCCAAGAACACGCTGTATCTGCAAATGAACAGTCTGAGAGCCGAGGACACGGCTGTGTATTACT
GTGCAAGAGA

Primer sequences

BIOMED-2		IG-NGS	
FR1	✓		
FR2	×		
FR3	✓	FR3	×

C Full nucleotide sequence clone IGH V3-74 -1/31/-10 J4

CGACAACGCCAAGAACACGCTGTATCTGCAAATGAACAGTCTGAGAGCCGAGGACACGGCTGTGTATTACTGTGCAA
GAGCTTAGTCGGGGAGTATAGCAACTCGCCCGGGCTACTGGGGCCAGGGAACCCT

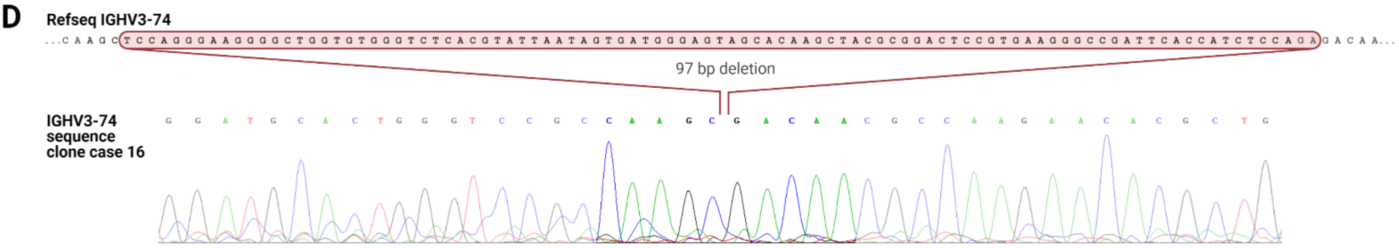

Supplementary Figure S7. Detection of a 97-bp deletion in IGHV region of classic Hodgkin lymphoma case 16. (A) The size of the clonal rearrangement detected with FR1 BIOMED-2/EuroClonality primers is located outside the expected size range for FR1 (231bp instead of 310-360 bp). In contrast, the clonal rearrangement detected with BIOMED2/EuroClonality FR3 primers fall within the expected size range of 100-170 bp, while FR2 primers failed to detect a clonal rearrangement (expected size range 250-295 bp). (B) Overview of the germline sequence of the IGHV3-74 gene (obtained from IMGT) together with the locations of the different primer binding sites (BIOMED-2/EuroClonality FR1 in green, FR2 in yellow, FR3 in red; IG-NGS FR3 in blue). The sequence in lower case indicate the 97 nucleotides that are deleted in the clonal rearrangement of case 16 as obtained by Sanger sequencing of the amplicon obtained with BIOMED-2 FR1 primers. Detection of the clone by the different primers is also indicated in the primer sequence legend. (C) Full nucleotide sequence of the clonal IGH V3-74 -1/31/-10 J4 rearrangement as detected by next-generation sequencing (IG-NGS). Bold sequence represents the V-gene sequence of this clonotype. (D) Sanger sequencing result for the FR1 clone. Top panel shows the region of the reference sequence of IGHV3-74 where the deletion (in red box) is located, the lower panel shows the actual Sanger sequencing result.

A Case 8

IGHV-IGHD-IGHJ (FR3)			IGHD-IGHJ			IGK - All targets
V3-9 -1/20/-6 J3	1.3%	Prod	D3-9 -3/33/-5 J6	2.6%	Unprod	Polyclonal
			D1-26 -2/8/-3 J5	1.5%	Unprod	

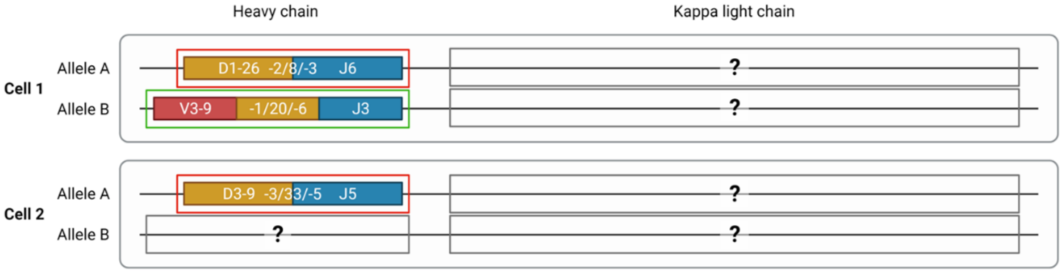

B Case 8

IGHV-IGHD-IGHJ (FR3)

	Clonotype	Junction aa sequence	Junction aa length	Abundancy (%)	Ratio over Background
1	**V3-9 -1/20/-6 J3**	**CAKDLSPDAGVFDVW**	**15**	**1,4984**	**4.95**
2	V4-39 -0/34/-2 J6	CARQTYSSGWYEKIVYYYYYGMDVW	25	0,3236	1.07
3	V4-59 -1/37/-2 J5	CASSGVYYYDSSGQENWFDPW	21	0,3095	1.02
4	V4-39 -2/8/-13 J6	CARVDGYGMDVW	12	0,3025	1.00
5	V4-34 -0/25/-5 J6	CARGIGGYSSSWYYYYGMDVW	21	0,3025	1.00
6	V4-39 -0/25/-2 J6	CAIQGYSYGYVQYYYYYGMDVW	22	0,3025	1.00
7	V4-39 -1/45/-9 J6	CARPSHVLL***WLLRIPNYYGMDVW	26	0,3025	1.00
8	V4-39 -0/38/-0 J4	CARHYKEGPGELLYLPYYFDYW	22	0,2955	0.98
9	V4-31=V4-34 -1/61/-0 J2	CARVQAGWADVYNNCSSTSCYTRVYWYFDLW	31	0,2955	0.98
10	V4-61 -0/21/-5 J6	CARLIYDSSSYYYYGMDVW	19	0,2814	0.93
11	V4-59 -2/22/-3 J2	CAKLRVNTSIWYFDLW	16	0,2744	0.91
12	V4-39 -3/29/-6 J6	CARDGTVAGTDWDYYYGMDVW	21	0,2673	0.88
13	V4-39 -0/14/-0 J4	CARQREVWNYDDYW	14	0,2533	0.84
14	V4-39 -0/26/-6 J5	CARHGHTIFGVVRFDPW	17	0,2533	0.84
15	V3-30=V3-30-3=V3-64 -0/40/-2 J6	CARDRVNYYDNTGYYKPYDYSHGMDVW	27	0,2533	0.84

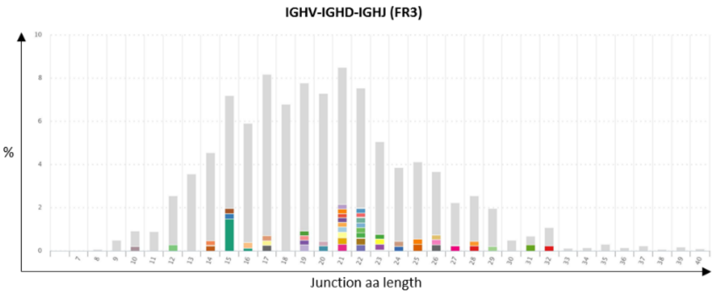

IGHD-IGHJ

	Clonotype	Junction aa sequence	Junction aa length	Abundancy (%)	Ratio over Background
1	**D3-9 -3/33/-5 J6**	**VVLRYFDWLL*VYCI*PVKPP#YYYYGMDVW**	**31**	**2,6164**	**8.51**
2	**D1-26 -2/8/-3 J5**	**VGIVGATSI##WFDPW**	**16**	**1,9529**	**6.35**
3	D3-3 -2/17/-0 J6	VVLRFLEWLL*GAICHYYSHYYGMDVW	27	0,3982	1.29
4	D3-9 -3/7/-3 J5	VVLRYFDWLLSP##WFDSW	19	0,3666	1.19
5	D3-9 -7/13/-5 J6	VLERCVDWLRNPF#YYYYGMDVW	23	0,3223	1.05
6	D3-22 -5/14/-4 J6	VVLL***WLFHGPN#YYYYGMDVW	24	0,3160	1.03
7	D3-3 -0/2/-0 J6	VVLRFLEWL*YR#YYYYYDMDVW	23	0,2844	0.92
8	D3-3 -5/12/-0 J6	VILRFLEWLLPPP##YYDYSVMDVW	25	0,2844	0.92
9	D3-22 -1/5/-7 J6	VVLL***WLLLKGYYYGMDVW	21	0,2781	0.90
10	D5-5=D5-18 -4/17/-1 J4	VVDTAMDTKTNH#YFDYW	18	0,2718	0.88
11	D3-3 -3/12/-15 J6	VLLRFLEWFLLLG*NSMDVW	20	0,2718	0.88
12	D3-22 -6/20/-11 J6	VVLL***WLSPNDPPGYYGMDVW	23	0,2718	0.88
13	D3-3 -6/17/-6 J6	VVLRFLEWLSRHIT*#YYYGMDVW	24	0,2718	0.88
14	D5-24 -2/5/-0 J4	VVEMATIY##YFDYW	15	0,2654	0.86
15	D5-24 -2/7/-3 J3	VVEMATI*G#AFDIW	15	0,2654	0.86

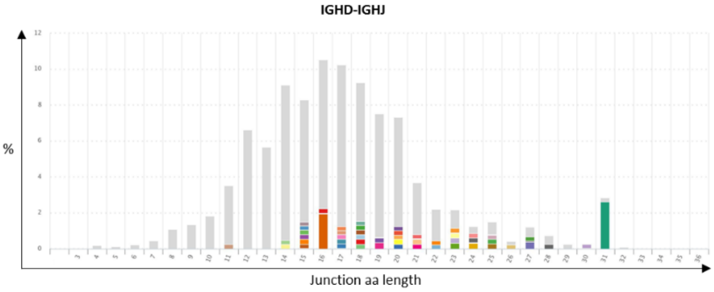

C Case 9

IGHV-IGHD-IGHJ (FR3)			IGKV-IGKJ			IGKV/intron RSS-KDE	
V4-34 -3/22/-3 J6	12.3%	Prod	V2-29 -4/0/-1 J3	5.6%	Unprod	Intron -2/1/-0 Kde	6.2%
V3-30=V3-33 -0/11/-0 J6	4.9%	Prod	V3-15=V3D-15 -0/2/-2 J1	4.9%	Prod	V2-28=V2D-28 -3/2/-0 Kde	6.0%
			V1-9 -2/1/-1 J4	4.7%	Unprod	Intron -4/3/-1 Kde	4.8%
						Intron -1/2/-4 Kde	4.6%

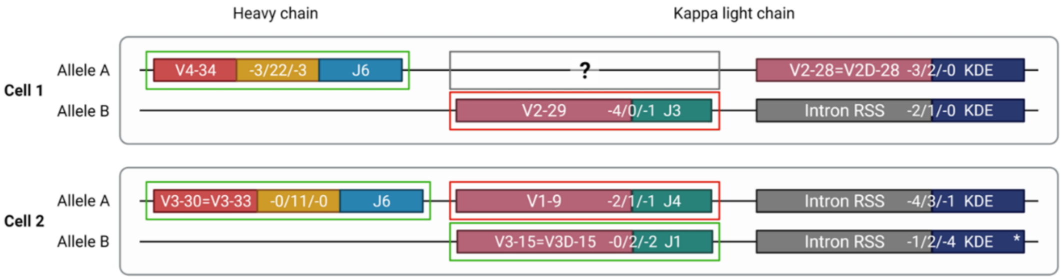

(Figure continues on next page)

D Case 9

IGHV-IGHD-IGHJ (FR3)

	Clonotype	Junction aa sequence	Junction aa length	Abundancy (%)	Ratio over Background
1	**V4-34 -3/22/-3 J6**	**CASTESYTFHYYYYGMDVW**	**19**	**15.104**	**28.36**
2	**V3-30=V3-33 -0/11/-0 J6**	**CARDTYGYYYVDYYGMDVW**	**19**	**4.0096**	**7.53**
3	V4-34 -2/23/-17 J6	CATYYYAPQPGMDVW	15	0.5728	1.08
4	V4-59 -1/10/-1 J6	CASAPD#CYYYYGMDVW	17	0.5728	1.08
5	V4-34 -0/21/-4 J5	CARGGSCSGGTCFDPW	16	0.5427	1.02
6	V4-61 -0/33/-5 J5	CARPNRESYSSSWAWFDPW	19	0.5427	1.02
7	V4-34 -0/34/-2 J4	CARGLGGGYDSSGYLYFDYW	20	0.5125	0.96
8	V4-31=V4-34 -1/29/-2 J4	CARVTPYCTNGVCYFDYW	18	0.5125	0.96
9	V2-5=V2-70 -0/27/-0 J4	CAHRREDWRWLQPYYFDYW	19	0.5125	0.96
10	V4-39 -0/34/-5 J5	CARHLRTPLIAHGVCWFDPW	20	0.4824	0.91
11	V3-49 -0/33/-0 J3	CTRDAPILYYYDSSAGAFDIW	21	0.4824	0.91
12	V4-59 -0/20/-0 J6	CARGGEQWLEYYYYYYGMDVW	21	0.4824	0.91
13	V4-34 -0/29/-7 J6	CARGLRFWSGPPV#YYYGMDVW	22	0.4824	0.91
14	V4-39 -0/31/-4 J6	CARHGGIQLWTYPR#YYYYGMDVW	24	0.4824	0.91
15	V3-64D -0/14/-0 J4	CVKDLHGTYSFDYW	14	0.4221	0.79

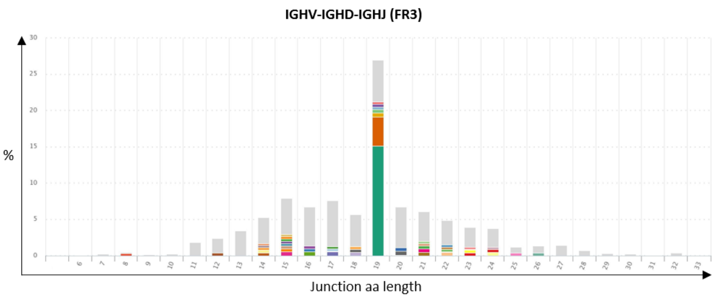

IGKV-IGKJ

	Clonotype	Junction aa sequence	Junction aa length	Abundancy (%)	Ratio over Background
1	**V2-29 -4/0/-1 J3**	***MQGIHLL#TF**	**11**	**6,9697**	**13.40**
2	**V3-15=V3D-15 -0/2/-2 J1**	**CQQYNNWPPGTF**	**12**	**3,7491**	**7.21**
3	**V1-9 -2/1/-1 J4**	**CQQLNSYP#LTF**	**12**	**2,7845**	**5.35**
4	V1-39=V1D-39 -3/0/-0 J4	CQQSYSTPLTF	11	0,6710	1.29
5	V4-1 -0/1/-5 J3	CQQYYSTPP#F	11	0,6123	1.18
6	V4-1 -3/0/-1 J2	CQQYYSTPYTF	11	0,5284	1.02
7	V1-39=V1D-39 -3/0/-1 J2	CQQSYSTPYTF	11	0,4194	0.81
8	V1-33=V1D-33 -1/0/-4 J4	CQQYDNLP#TF	11	0,3774	0.73
9	V2-29 -7/4/-0 J1	*MQGIHPPWTF	11	0,3355	0.65
10	V2-30=V2D-30 -1/0/-4 J4	CLQGAHWP#TF	11	0,3355	0.65
11	V2D-26 -2/2/-0 J1	CMQDAQDPGWTF	12	0,3355	0.65
12	V2-29 -7/3/-0 J5	*MQGIHP#ITF	11	0,3271	0.63
13	V2D-26 -2/3/-2 J5	CMQDAQDPW#TF	12	0,3271	0.63
14	V1-39=V1D-39 -1/0/-2 J4	CQQSYSTPLTF	11	0,3187	0.61
15	V3-20=V3D-20 -3/0/-1 J2	CQQYGSSPYTF	11	0,3103	0.60

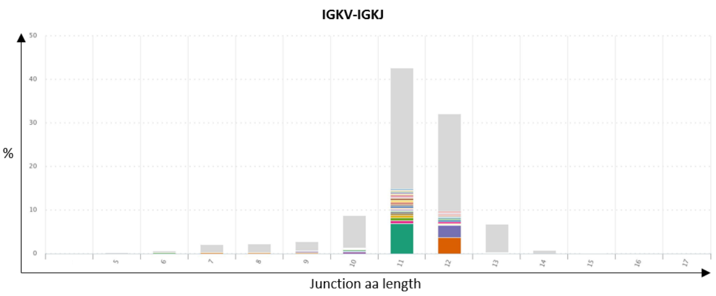

IGKV/Intron RSS-KDE

	Clonotype	Junction aa sequence	Junction aa length	Abundancy (%)	Ratio / %
1	**intron -2/1/-0 Kde**	**PCVCPINAAVASFPD#SPSGSPGR**	**24**	**7.3361**	**7.34%**
2	**V2-28=V2D-28 -3/2/-0 Kde**	**CMQALQTPRSPSGSPGR**	**17**	**7.1800**	**4.50**
3	**intron -1/2/-4 Kde**	**PCVCPINAAVASFPDNPSGSPGR**	**23**	**5.9573**	**5.96%**
4	**intron -4/3/-1 Kde**	**PCVCPINAAVASFPS#SPNGSPGR**	**24**	**5.1769**	**5.17%**
5	intron -0/0/-2 Kde	PCVCPINAAVASFPD#SPSGSPGR	24	2.0031	2.00%
6	intron -2/0/-3 Kde	PCVCPINAAVASFP##PSGSPGR	23	1.7170	1.72%
7	V2-29 -3/2/-1 Kde	*MQGIHLP#SPSGSPGR	17	1.0666	0.67
8	V1-39=V1D-39 -3/0/-0 Kde	CQQSYST##SPSGSPGR	17	1.0406	0.65
9	intron -0/0/-4 Kde	PCVCPINAAVASFPD#PSGSPGR	23	0.9625	0.96%
10	intron -3/3/-7 Kde	PCVCPINAAVASFPA#SGSPGR	22	0.9625	0.96%
11	V7-3 -3/5/-3 Kde	CLQSKNFPQGPSGSPGR	17	0.8845	0.55
12	intron -1/4/-5 Kde	PCVCPINAAVASFPDR#PSGSPGR	24	0.7544	0.75%
13	V1-37=V1D-37 -4/4/-4 Kde	GQRTYNALLPSGSPGR	16	0.7544	0.47
14	intron -1/0/-8 Kde	PCVCPINAAVASFPDSGSPGR	21	0.7284	0.73%
15	intron -1/1/-6 Kde	PCVCPINAAVASFPDSSGSPGR	22	0.7024	0.70%

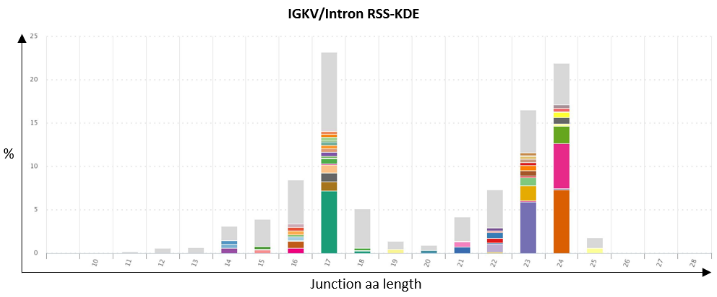

Supplementary Figure S8. Detection of biclonal classic Hodgkin lymphoma. (A, C) Hypothetical scenario for the distribution of clonal gene rearrangements over two different HRS cells are shown for case 8 (A) and case 9 (C). The upper panel shows all detected clonal IGH and IGK rearrangements with their mean abundancy and predicted productivity. For case 8, the clonotypes and their percentages are based on combined results from FF and FFPE, for case 9 this is based on FF results only. The lower panels show the schematic overview of the possible distribution of the clonotypes over two different HRS cells. Productivity is indicated by green and red boxes for productive and unproductive rearrangements, respectively. *Although the IGKV-IGKJ rearrangement on this allele is predicted as a productive rearrangement, the rearrangement involving KDE could still be present on the same allele when the IGKV-IGKJ rearrangement results in a self-reactive BCR, followed by receptor editing and Intron RSS-KDE recombination. (B, D) Additional information of the top-15 most abundant clonotypes (table) and corresponding bar-chart (complete dataset) of representative analyses are shown for targets with clonal rearrangements. All clonal rearrangements have ratio's above defined thresholds for clonality (IGHV-IGHD-IGHJ (FR3): 2.5 ; IGHD-IGHJ: 4.5 ; IGKV-IGKJ: 4.0 ; IGKV-KDE: 2.5), or an abundancy above 4.5% (Intron RSS-KDE threshold for clonality), and are shown in bold. The colors indicated in the table correspond to the colors of the bars in the bar-chart. On the x-axis, the junction amino acid (aa) length is shown; on the y-axis the abundancy of detected clonotypes in percentage. Each bar shows all clonotypes with the same junction aa length and each color represents a unique clonotype. The top-50 most abundant clonotypes are colored, all other clonotypes are represented by the grey bars. (B) Case 8: IGHV-IGHD-IGHJ (FR3) and IGHD-IGHJ results are based on a representative, single analysis of FF or FFPE DNA, respectively. (D) Case 9: IGHV-IGHD-IGHJ (FR3), IGKV-IGKJ and IGKV/Intron RSS-KDE results are shown, all based on a representative single analysis of FF DNA.

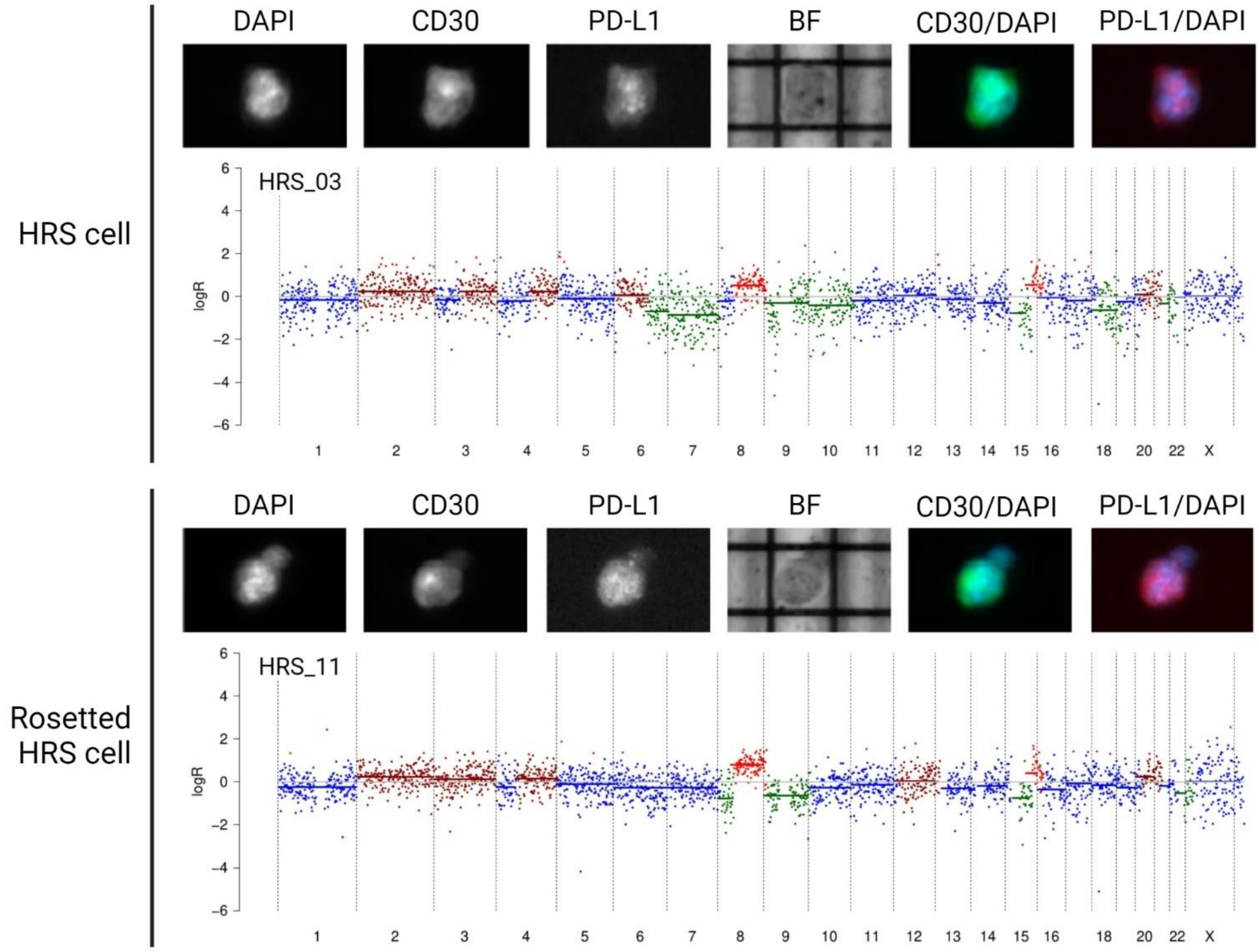

Supplementary Figure S9. Microscopy images and corresponding copy number variation (CNV) profiles of a single HRS cell and a T cell-rosetted HRS cell. The CNV profiles show the genome location according to chromosome position on the x-axis and the log-scaled copy number values on the y-axis. Both profiles are from cells in cluster 2 and show no significant differences caused by the rosetted T cell.

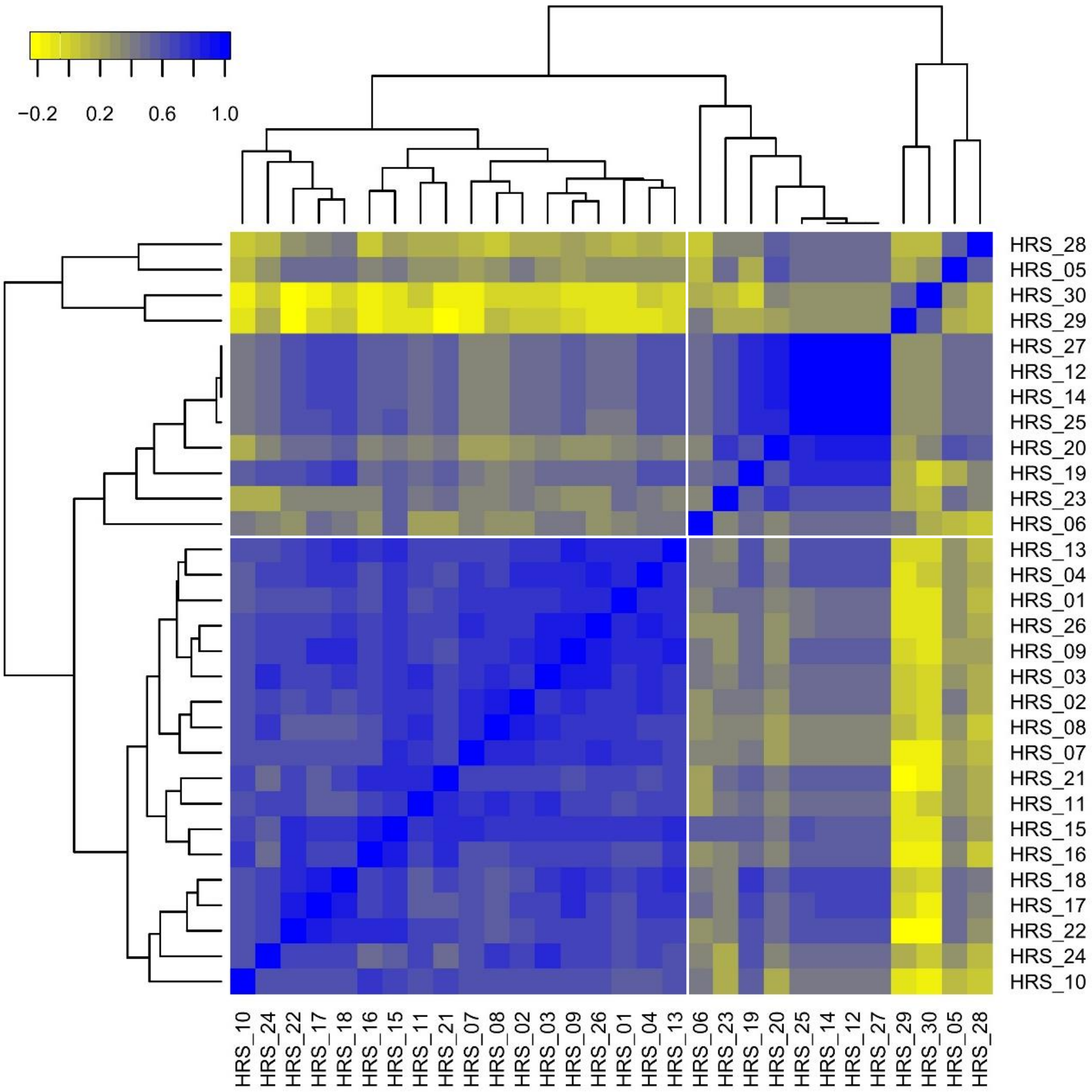

Supplementary Figure S10. Cell-to-cell correlation plot of the CNV profiles of digital sorted HRS cells demonstrates two main populations of tumor cells displaying intraclonal heterogeneity. CNV profiles of the different HRS cells analyzed after DEPArray digital sorting technology on the basis of CD30 and PD-L1 expression are correlated to each other. Each analyzed HRS cell is shown on both the x- and y-axis to compare all cells with each other. A perfect cell-to-cell correlation is shown in bright blue. Samples that are different from each other are indicated in bright yellow, intermediate phenotypes are proportionally indicated with a gradient towards yellow. Unsupervised hierarchical clustering reveals a main homogeneous cluster at the lower left corner. Although the clustering-arms show that the cells in the top right corner belong to two separate branches, these cells still form a single but heterogeneous cluster when the cluster results of the CNV profiles is taken into account. So there are two main clusters that may be indicative for biclonality, and that show the presence of intraclonal diversity within each population of tumor cells.

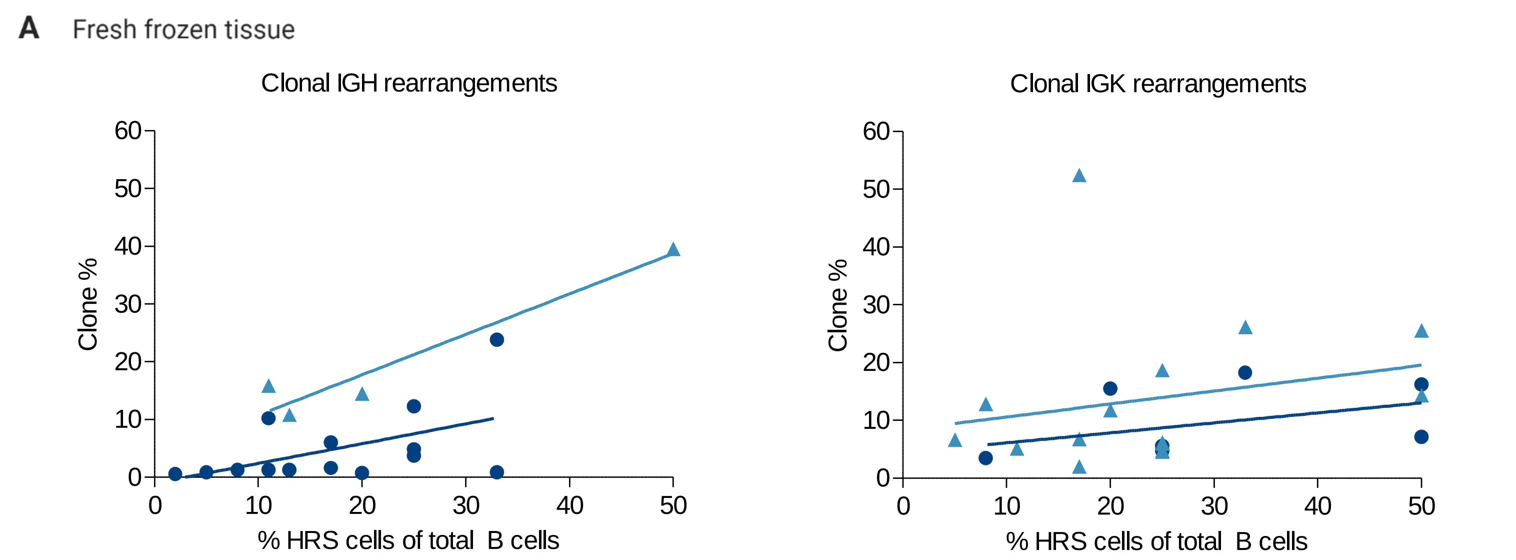

	Spearman Rank	P-value
IGHV-IGHD-IGHJ (FR3)	0.4939	0.0726
IGHD-IGHJ	0.4000	0.7500

	Spearman Rank	P-value
IGKV-IGKJ	0.6138	0.1150
IGKV/Intron RSS-KDE	0.2631	0.3435

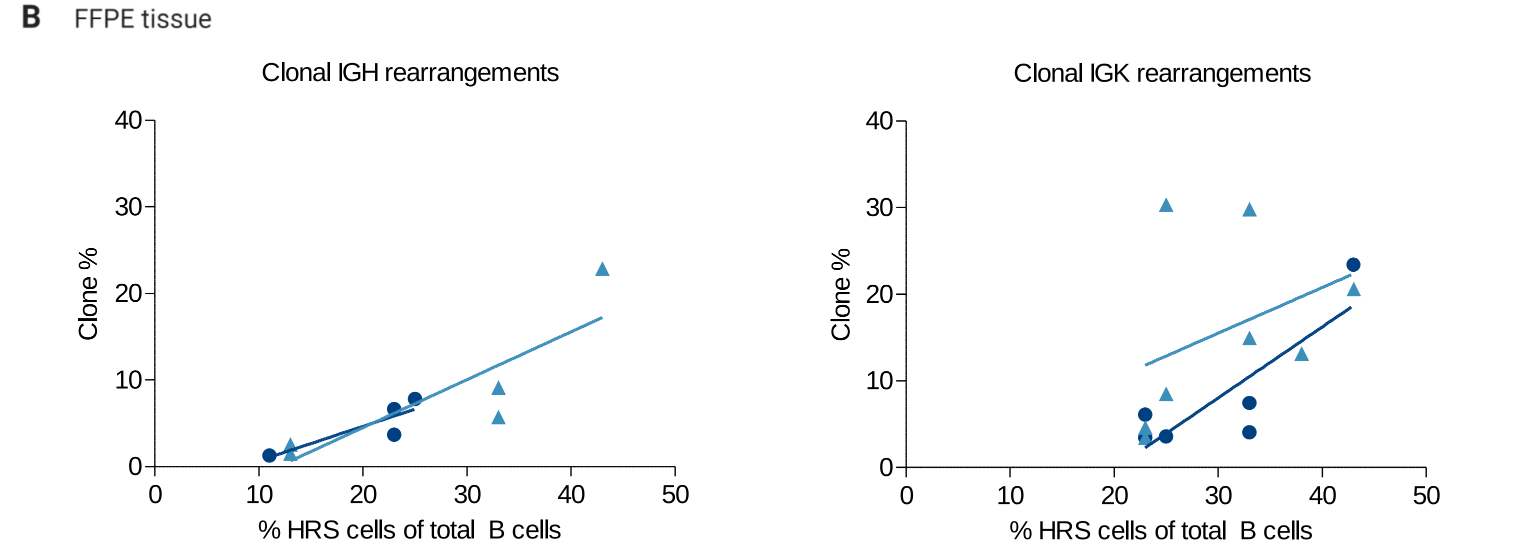

	Spearman Rank	P-value
IGHV-IGHD-IGHJ (FR3)	0.9487	0.0833
IGHD-IGHJ	0.9487	0.0167*

	Spearman Rank	P-value
IGKV-IGKJ	0.7062	0.1361
IGKV/Intron RSS-KDE	0.5577	0.1511

Supplementary Figure S11. Correlation between the percentage of HRS cells of total B cells and the percentage of clonal rearrangements. For each clonal rearrangement on the heavy chain (IGH, left panels) and kappa light chain (IGK, right panels), the relative amount of HRS cells and the clone size are shown on the x- and y-axis respectively. A linear regression line is shown per target (IGHV-IGHD-IGHJ (FR3) and IGHD-IGHJ or IGKV-IGKJ and IGKV/Intron RSS-KDE), as well as the Spearman Rank with P-value. Results are shown for fresh frozen tissue (A), or FFPE tissue (B).

Supplementary Table S6.

Productivity of clonal IGHV-IGHD-IGHJ rearrangements per case.

Case	Clonotype	CDR3 aa sequence	CDR3 aa length	Productivity*
**1**	V3-48=V3-69-1 -0/47/-3 J6	CTRDLSSLNQSESTGFYSVDHSYYGMDVW	29	Productive
**2**	V1-3 -0/26/-9 J6	CARESMGVNFLRDSFALDVW	20	Productive
**3**	V4-59 -1/56/-6 J6	CASVVPAAIT*SGLL*PPKPADYYYGMDVW	30	Unproductive
**4**	Polyclonal	-	-	-
**5**	V3-48=V3-69-1 -3/31/-1 J3	CARRGILGDYDSFDAFDVW	19	Productive
**6**	Polyclonal	-	-	-
**7**	~V3 -4/20/-2 J4	CAIYSGHHRYFDSW	14	Productive
**8**	V3-9 -1/20/-6 J3	CAKDLSPDAGVFDVW	15	Productive
**9**	V4-34 -3/22/-3 J6	CASTESYTFHYYYYGMDVW	19	Productive
	V3-30=V3-33 -0/11/-0 J6	CARDTYGYYYVDYYGMDVW	19	Productive
**10**	V3-71 -6/17/-0 J6	CATTALGNYYHFNGLDFW	18	Productive
**11**	V3-23=V3-23D -0/26/-6 J6	CAKDPGYSYGLRDYYYGMDTW	21	Productive
**12**	V3-9 -2/25/-2 J4	CVKEVDVDTVKHYFDYW	17	Productive#
	V3-30 -5/33/-0 J6	CVKGGPHDYDDRDHSFHYGMDVW	23	Productive#
**13**	V3-74 -1/60/-4 J6	CTSTGAAQEVFGDSYDSSPYYATYYYYGMDVW	32	Productive
	V2-26 -0/21/-5 J6	CARIQTAAAGM#YYYYGMDVW	21	Unproductive
**14**	Polyclonal	-	-	-
**15**	Polyclonal	-	-	-
**16**	Polyclonal	-	-	-

* Productivity of the clonotypes is predicted based on an in-frame CDR3 sequence. # Based on the FR3 results the presence of an upstream frameshift resulting in an unproductive B-cell receptor cannot be excluded. Therefore this sample is not marked as a biclonal case in the study. CDR3: complementarity-determining region 3; aa: amino acid.

Supplementary References

1 van Dongen JJ, Langerak AW, Bruggemann M, Evans PA, Hummel M, Lavender FL, et al. Design and standardization of PCR primers and protocols for detection of clonal immunoglobulin and T-cell receptor gene recombinations in suspect lymphoproliferations: report of the BIOMED-2 Concerted Action BMH4-CT98-3936. *Leukemia* **17**, 2257-2317 (2003).

2 Langerak AW, Groenen PJ, Bruggemann M, Beldjord K, Bellan C, Bonello L, et al. EuroClonality/BIOMED-2 guidelines for interpretation and reporting of Ig/TCR clonality testing in suspected lymphoproliferations. *Leukemia* **26**, 2159-2171 (2012).

3 van den Brand M, Rijntjes J, Mobs M, Steinhilber J, van der Klift MY, Heezen KC, et al. Next-Generation Sequencing-Based Clonality Assessment of Ig Gene Rearrangements: A Multicenter Validation Study by EuroClonality-NGS. *J Mol Diagn* **23**, 1105-1115 (2021).

4 Juskevicius D, Jucker D, Dietsche T, Perrina V, Rufle A, Ruiz C, et al. Novel cell enrichment technique for robust genetic analysis of archival classical Hodgkin lymphoma tissues. *Lab Invest* **98**, 1487-1499 (2018).

5 Mangano C, Ferrarini A, Forcato C, Garonzi M, Tononi P, Lanzellotto R, et al. Precise detection of genomic imbalances at single-cell resolution reveals intra-patient heterogeneity in Hodgkin's lymphoma. *Blood Cancer J* **9**, 92 (2019).

6 Adalsteinsson VA, Ha G, Freeman SS, Choudhury AD, Stover DG, Parsons HA, et al. Scalable whole-exome sequencing of cell-free DNA reveals high concordance with metastatic tumors. *Nat Commun* **8**, 1324 (2017).
